# Goal-related feedback guides motor exploration and redundancy resolution in human motor skill acquisition

**DOI:** 10.1371/journal.pcbi.1006676

**Published:** 2019-03-05

**Authors:** Marieke Rohde, Kenichi Narioka, Jochen J. Steil, Lina K. Klein, Marc O. Ernst

**Affiliations:** 1 Institute for Innovation and Technology (IIT) Berlin, Germany; 2 Center of Excellence: Cognitive Interaction Technology (CITEC), University of Bielefeld, Bielefeld, Germany; 3 DENSO Corporation, Tokyo, Japan; 4 Insitute for Robotics and Process Control, Technische Universität Braunschweig, Braunschweig, Germany; 5 Department of Psychology, University of Giessen, Giessen, Germany; 6 Department of Applied Cognitive Psychology, University of Ulm, Ulm, Germany; New York University, UNITED STATES

## Abstract

The plasticity of the human nervous system allows us to acquire an open-ended repository of sensorimotor skills in adulthood, such as the mastery of tools, musical instruments or sports. How novel sensorimotor skills are learned from scratch is yet largely unknown. In particular, the so-called inverse mapping from goal states to motor states is underdetermined because a goal can often be achieved by many different movements (motor redundancy). How humans learn to resolve motor redundancy and by which principles they explore high-dimensional motor spaces has hardly been investigated. To study this question, we trained human participants in an unfamiliar and redundant visually-guided manual control task. We qualitatively compare the experimental results with simulation results from a population of artificial agents that learned the same task by *Goal Babbling*, which is an inverse-model learning approach for robotics. In Goal Babbling, goal-related feedback guides motor exploration and thereby enables robots to learn an inverse model directly from scratch, without having to learn a forward model first. In the human experiment, we tested whether different initial conditions (starting positions of the hand) influence the acquisition of motor synergies, which we identified by Principal Component Analysis in the motor space. The results show that the human participants’ solutions are spatially biased towards the different starting positions in motor space and are marked by a gradual co-learning of synergies and task success, similar to the dynamics of motor learning by Goal Babbling. However, there are also differences between human learning and the Goal Babbling simulations, as humans tend to predominantly use Degrees of Freedom that do not have a large effect on the hand position, whereas in Goal Babbling, Degrees of Freedom with a large effect on hand position are used predominantly. We conclude that humans use goal-related feedback to constrain motor exploration and resolve motor redundancy when learning a new sensorimotor mapping, but in a manner that differs from the current implementation of Goal Babbling due to different constraints on motor exploration.

## Author summary

Even in adulthood, humans can learn to master new motor skills with unfamiliar mappings between desired goals or sensations and corresponding movements, such as playing tennis or musical instruments. To master a new skill involves the resolution of *motor redundancy*; that is a selection from many possible movements that all achieve the same goal. Here, we trained participants in a redundant and unfamiliar task that mapped their hand movements to shapes, in order to investigate which of the many possible redundant solution participants learn. The results show that local task feedback, which depends on the starting posture of the hand, influences participants’ motor learning. We qualitatively compared the experimental results to computer simulations of artificial agents that learned the same task by Goal Babbling, i.e., a motor learning approach used in robotics, to assess if humans might learn the task following similar principles. Both the simulated agents and the participants show sensitivity to goal-directed feedback during learning, but they use different strategies to explore the movement space. We conclude that human motor learning and redundancy resolution is guided by local goal feedback, like in Goal Babbling, but that differences in motor exploration lead to different learning outcomes.

## Introduction

How humans acquire novel sensorimotor skills from scratch is one of the big open questions in motor control. Most of the literature on motor plasticity focuses on the *adaptation* of motor control: An already existing pattern of behaviour is perturbed, e.g., by introducing displacements, rotations, force perturbations or delays into the mapping from actions to sensory feedback. It is then studied how humans compensate for the perturbation. Computational models of such motor adaptation usually assume the previous existence of a motor control policy that is then recalibrated by motor adaptation [1,2,3].

Sometimes, humans have to learn a novel sensorimotor mapping from scratch, e.g., during development or when learning to use a new tool, musical instrument or sporting equipment. Such processes of *motor acquisition* [1,4] require setting up a new control policy based on an *inverse model* [5]. That is, a new mapping has to be learned to map goals in a *goal space*, e.g., Cartesian coordinates that parameterize throwing, grasping or reaching targets, to states in the *motor space*, e.g., muscle activation patterns or joint configurations that achieve this goal. Some models of motor learning assume that this requires learning the forward model from motor actions to goals as an intermediate step [5]. However, also a *direct* learning of inverse models without this intermediate step has been considered as a possible mechanism. In both cases, the presumed mechanisms of motor learning differ from those used for motor adaptation [6].

The skills learned in motor acquisition are often non-linear, high-dimensional and *redundant* [7]. That is, the inverse mappings from states in the goal space to motor commands are underdetermined: I can pick up a cup (one goal) with my arm extended, with my arm flexed, with the palm pointing downwards, the palm pointing sideways, with my left arm, my right arm, or even my mouth (one-to-many goal-to-movement mapping). We do not know how motor redundancy is resolved during learning and how this in turn influences the learning dynamics.

We here present results from an experiment to investigate the role of goal-related feedback and local exploratory activity in motor redundancy resolution during motor skill acquisition. Human participants were trained in an unfamiliar and redundant visually-guided manual control task, starting from different initial states. We qualitatively compare the experimental results to computer simulations of a population of artificial agents that learn the task by Goal Babbling, which is an inverse model learning approach in robotics [8,9]. The following sections relate our approach to the relevant literature in robotics, motor control, and motor development before the hypothesis is presented.

### Human development, Motor Babbling, and Goal Babbling

The paradigmatic example of motor acquisition is infant motor development. Humans are born without a complete innate knowledge of how their bodies are configured. They lack motor coordination and have to learn how to control their hands, heads, feet, etc., by exploratory activity. It has been observed that infants undergo a phase in their development during which they seem to perform random movements [10,11]. Piaget’s view was that motor development is organized in distinct phases and that this presumed random movement phase might serve to learn an inverse kinematic model of the body. That is, he suggested that during this random movement learning phase, infants do not perform purposeful actions. According to this hypothesis, only in a second phase, when the inverse model has been learned, infants start performing goal-directed action [10].

This idea has inspired learning algorithms in Cognitive and Developmental Robotics. From a computational perspective, the problem of inverse motor model learning can be seen as a search problem, i.e., the search for a control policy or inverse model that links the behavioural goals (states in goal space) to available actions (states in motor space). So-called *Motor Babbling* algorithms [e.g., 12] solve this problem in a very straightforward, brute-force manner inspired by the traditional view on infant development described above: In an initial ‘infant’ stage, robots perform random actions (Motor Babbling) to uniformly and exhaustively sample the motor space. They observe and memorize the consequences of the action to derive a generalized rule for action-outcome mappings (forward model). This model can then, in a second phase, be inverted (at least locally) for control. The resulting inverse model is used to perform goal-directed actions in the ‘adult’ robot.

Motor Babbling algorithms have also been proposed as generative models of infant motor acquisition [12,13], in line with Piaget’s stage-wise view, which furthermore is compatible with the traditional view on computational inverse model learning, i.e., that a feedforward model has to exist first to acquire an inverse model second [14,15]. However, in human development, Piaget’s view that motor learning is prevalently driven by random exploration has been criticized [11] in view of empirical evidence that infants perform goal-directed action right from the outset of motor learning [11,16], which results in complicated learning dynamics, as it has been shown in elaborate experiments [17]. Furthermore, it has been observed that the first successful reaching actions in infants appear to be executed fully feedforward [18,19], which would require the existence of an inverse model to start with. These results speak against the traditional view of a stage-wise motor acquisition process with a random exploration phase (forward model learning) followed by a phase of inverse model generation and purposeful action, and in favour of a direct acquisition of the inverse model while behaving.

Goal Babbling is a computational approach inspired by this latter idea that infant motor acquisition might be goal-directed right from the start. It has recently been proposed by Rolf, Steil & Gienger [8,9] as a model for learning of an inverse model while behaving (direct inverse model learning). Thereby, the exhaustive random sampling phase characteristic of Motor Babbling can be avoided and there is no need to distinguish explicit data collection and exploitation phases anymore. Related goal directed exploration schemes for motor learning that share the idea to guide exploration by goal-related feedback have also been proposed by others [20].

In Goal Babbling, a robot’s exploration of the motor space is not just random. Instead, it is constrained and guided by goals. A starting position (home posture) seeds the search for a successful policy. The expansion of the motor repertoire around the home posture occurs through random perturbations that are reinforced in the direction that is most associated with behavioural success: Goal-directed feedback (reinforcement) favours the future use of actions that are associated with a reduction in a goal-related error signal.

A robot thereby locally expands its action repertoire around an initially minimal inverse model control policy that maps all goals onto a single motor command, which keeps the robot resting at the home posture. Goal-irrelevant parts of the motor space or parts that contain solutions that are redundant to the initially learned one are thus never explored (see Method Sect Goal Babbling Simulations and [8,9] for details of the Goal Babbling algorithm).

In a reinforcement learning context, Goal Babbling for inverse model learning can be seen as a one-step reinforcement learning problem, where distance to the goal can be reinterpreted as a reward [21], but for a very large number of goals, which distinguishes inverse model learning from standard reinforcement learning problems. Standard episodic reinforcement learning algorithms, for instance, aim at learning a temporal control policy to solve a particular defined task or task sequence with a potentially distant reward. Many successful computational algorithms for this type of problem have been proposed in recent years [22]. They are heavily applied in robotics and have been linked to human reinforcement learning. However, these reinforcement learning algorithms, though related to Goal Babbling, are not easily applicable to the problem of direct inverse model learning from scratch discussed here. There is ample evidence [5] that (adult) human motor control relies on both forward and inverse models and the question how inverse models are learned is an open research question.

Other computational models for learning inverse models online have been proposed recently, but again require the prior existence of a working feedback controller (see the review in [23]). In all these approaches, additional measures have to be taken to deal with redundancy in the action-goal mapping. In summary, neither standard theories of model learning nor (episodic) reinforcement tackles the problem of acquiring an inverse model for a novel sensorimotor mapping in a redundant domain that we focus on here.

In the robotics domain, Goal Babbling is particularly useful for platforms that, like humans, have many and redundant Degrees of Freedom (DoFs) with non-linear dependencies between them [24]. Other inverse model learning approaches like Motor Babbling struggle to learn the inverse models for such platforms. Goal Babbling as a model for infant motor skill acquisition [25] has already been shown to reproduce the dynamics of the so-called U-shaped sequence of disappearance and reappearance of a skill for pre-reaching to balls presented to infants of early age [17]. Here, we study Goal Babbling as a computational model for adult human motor learning. The experiment we present here investigates whether learning dynamics similar to Goal Babbling might occur when humans learn an unfamiliar and redundant sensorimotor mapping from scratch: It tests for Goal Babbling-like motor exploration (i.e., local goal-directed expansion of the action repertoire, no distinction between data collection and exploitation phases) in a motor skill acquisition task with adult volunteers (see Introduction Sects Motor Skill Acquisition in Human Adults and Do adult humans use error feedback to guide motor skill acquisition?).

### Motor skill acquisition in human adults

Human adults who have already mastered control over their bodies can also learn new high-dimensional and redundant motor tasks, when interacting with objects such as musical instruments, tools, toys, or sports equipment. This kind of motor skill acquisition differs from motor development in the sense that it involves adding a new layer *on top* of the already familiar control of one’s body. However, the requirements posed on the neural mechanisms of learning are similar: The interaction with the object brings about sensory effects in response to motor actions, such as sounds when playing instruments, forces when operating tools, or visual effects when playing computer games. The rules that connect sensory effects with movements are initially unfamiliar. To master control of the interaction, humans have to explore the oftentimes high-dimensional and non-linear mapping from states in motor space to states in goal space and to constrain and memorize the non-unique inverse mapping from goal states to motor states, much like during infant motor development. We here test whether Goal Babbling simulations can qualitatively predict the dynamics and outcome of motor skill acquisition in adult humans, which would suggest that humans might use similar principles for motor exploration and inverse model learning.

It has been suggested that motor skill acquisition involves the formation of *Motor Synergies*, i.e., of patterns of DoF co-activation that can serve as motor primitives and reduce the dimensionality of a motor control problem [7,26]. In many tasks that involve redundant DoFs, motor behaviour has been shown to be reducible to the linear combination of just two or three spatio-temporal movement components (synergies), for instance, kicking behaviour in frogs [27], human object grasping [28] or even full body walking and reaching [29]. The question of redundancy resolution can therefore also be understood as a question of synergy formation. We here use this approach, that is, we measure motor organization and dimensionality reduction by synergy formation, which we define as principal components in the motor space (see Method Sect Data Analysis). This approach has also been previously used to describe redundancy resolution in robotic Goal Babbling [30].

### Do adult humans use error feedback to guide motor skill acquisition?

The focus of our work is on human adult learning of a new, redundant inverse sensorimotor mapping from goals to actions. A number of principles have been shown to influence motor learning of redundant motor control problems. For instance, participants adapt behaviour optimally according to simple, goal-related cost functions [2, 31] as well as to achieve dynamic stability of control [32, 33]. Here, we were interested primarily in principles of motor exploration and whether they follow more the traditional view (which we here call “Motor Babbling”), where a random exploration phase is followed by a goal-directed action phase, or the integrated process view that underlies the Goal Babbling approach. Do humans use goal-related feedback right from the start to guide the exploration of a motor space and the continuous formation of synergies as in Goal Babbling? In particular, we were interested in testing the following predictions from Goal Babbling:

P1. *Synergies are learned gradually during the course of the learning dynamics (motor organization)*.
We here define synergies as a subset of motor Principle Components that can explain most of the variance in motor space (dimensionality reduction) and that represent DoF co-activation patterns [30] (Results Sect Motor Synergy Formation, Methods Sect Postures in Motor Space (q_1_, q_2_, q_3_)). Goal Babbling predicts the gradual formation of synergies, Motor Babbling predicts a phase of random exploration (no synergies) followed by a phase of goal-directed action (possible transition to using synergies).
P2.*The inverse model gradually unfolds from the home posture (starting condition) in motor space and is biased towards its location*.
Comparing two conditions with different home postures, Goal Babbling predicts that the learned solutions will expand away from the home posture and might remain biased towards their location (Results Sect Location of solutions). Motor Babbling suggests exhaustive sampling of the motor space and thus predicts no such bias.
P3. *If local movement of a particular DoF is associated with partial task success*, *this DoF will be used more for exploration*.
We manipulate the local effectivity of the DoFs by choice of the home posture in the two conditions. Goal Babbling predicts that a DoF is used more for action in the condition where it is more locally effective (we measure this by its contribution to the first synergy PC1, Results Sect Relative use of DoFs in synergies). Motor Babbling predicts no differences in use, as exploration is deemed random.

In summary, Motor Babbling predicts distinguishable phases of exploration and goal-directed behaviour as well as learning outcomes that do not depend on the condition (home posture). Goal Babbling predicts an integrated exploration, learning and goal-directed action process, where task-related feedback for action guides and biases the learning process and outcome for inverse model learning.

**Fig 1:**
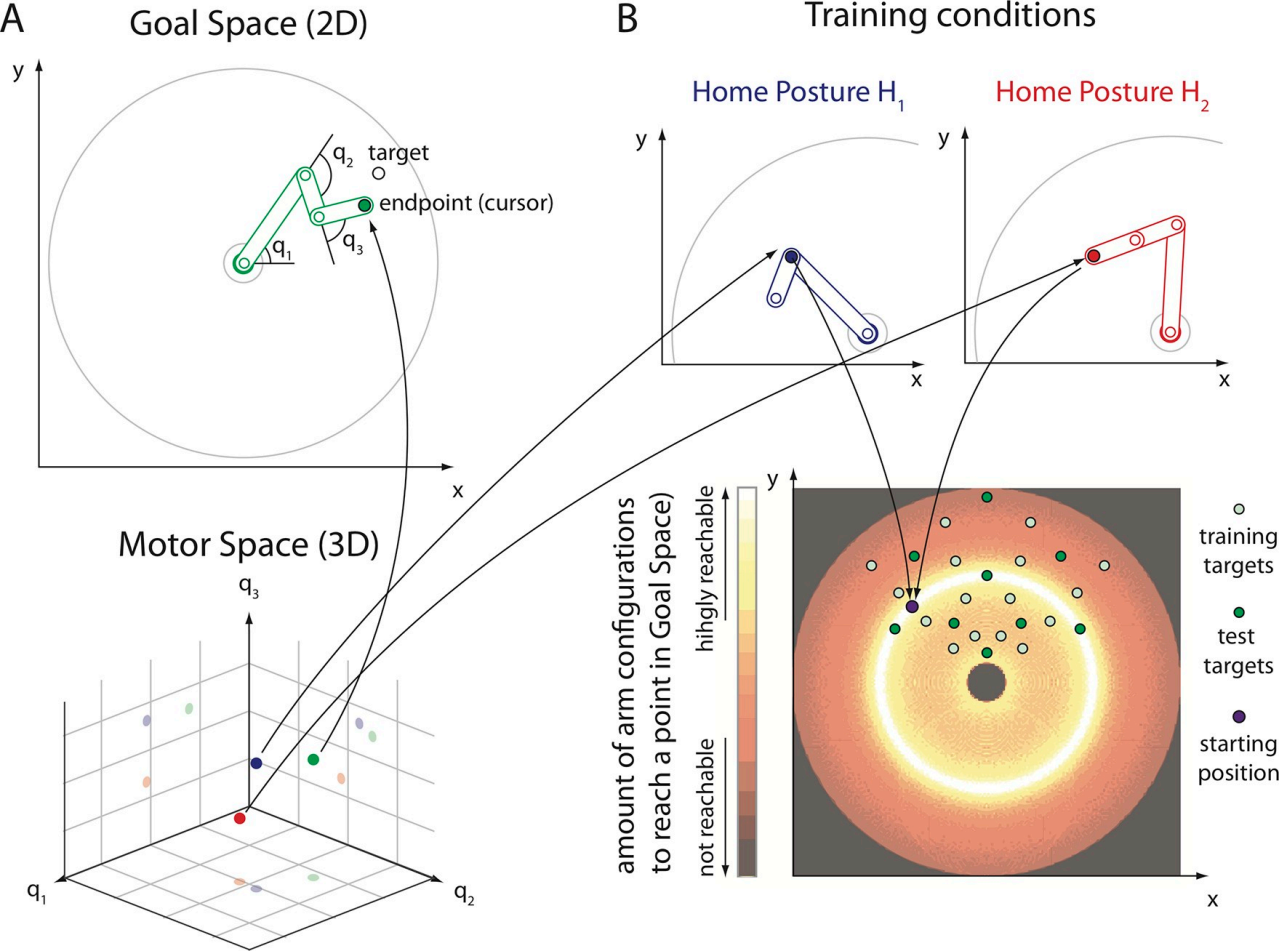
The three DoF planar arm reaching task. **A:** Both humans and simulated agents learned to move a three DoF planar arm with joints *q*_*1*_, *q*_*2*_ and *q*_*3*_ (three-dimensional motor space) to reach to targets in an *x* and *y* plane (two-dimensional goal space). **B**: Top: The home postures *H*_*1*_ and *H*_*2*_ are different but reach to the same point in goal space. Note that in home posture *H*_*1*_, the *q*_*2*_ joint is folded backwards (*q*_*2*_ = π) so the reach-endpoint is located on top of the *q*_*2*_ joint. Bottom: The colour map depicts the redundancy, that is the relative number of joint configurations *(q*_*1*_, *q*_*2*_, *q*_*3*_*)* that reach to a given point in the goal space (lighter colours imply more joint configurations to reach this point). The task is redundant, especially for targets close to the origin of the goal space. Participants were trained and tested on targets in the top quadrant of the goal space (green disks).

To test these predictions, we trained human participants in an unfamiliar and redundant sensorimotor control task (Fig 1 and Method Sects Task and Experimental Setup and Procedure). We also qualitatively compared the results to those of a population of simulated agents that were trained on the same task using the Goal Babbling learning approach (Method Sects Goal Babbling Simulations), to illustrate the Goal Babbling predictions more concretely. For these agents, the task was to set the joint angles of a three DoF planar arm (Fig 1A) to reach to targets in a quarter segment of the circular reach space (Fig 1B, bottom, and Method Sect Task). Human participants were trained in the same task but the input variables were presented in an unfamiliar transformation to avoid that participants might rely on prior knowledge, e.g., about physics or joint kinematics, which can facilitate motor learning [34]. Participants therefore controlled the joint angles of the task by the elevation of the left index, right index, and right middle fingers, unaware that this was internally interpreted as joint angle settings (Method Sect Experimental Setup and Procedure).

Moreover, the positions of targets and reach-endpoints in goal space were presented as static, deforming ellipses (Method Sect Experimental Setup and Procedure) to avoid that the solution learned is constrained by principles of spatial processing, as it is the case in human motor skill acquisition of spatial control tasks. For instance, Mosier et al. [35] have shown that participants tend to learn solutions that generate rectilinear movements of a cursor in visual space. Sailer et al. [36] have investigated hand-eye coordination in a similar task and have found that with increasing mastery of the task, participants first learned to perform predictive eye movements and later saccades to a target, which demonstrates remapping not only between hand movements and cursor movements but also alignment with oculomotor control. By presenting the targets and reach-endpoints as static, deforming ellipses, we aimed at reducing such biases inherent to spatial tasks. In summary, the mapping from finger movements to elliptic shapes was, mathematically speaking, that of reaching with a three DoF planar arm (Fig 1). However, participants experienced this as deforming ellipses with up-down finger movements (Method Sect Experimental Setup and Procedure).

The task we chose is redundant: There are infinitely many DoF configurations to reach each target. Especially targets that are close to the origin of the goal space can be reached from many parts of the motor space (Fig 1B, bottom). Note that the degree of redundancy, here measured as number of different configurations to reach a particular target in a discretized version of the task is non-homogeneous and strongly varying across the target space (Fig 1B, bottom, colour coding). We analysed redundancy resolution by performing Principal Component Analysis (PCA) on the reach-endpoints in motor space in a test phase with no feedback. Thereby, we could identify DoF co-activation patterns (synergies) in the learned inverse mappings from goal states to motor states that can reduce the dimensionality of a complex, redundant control problem and are a sign of motor organization (Method Sect Data Analysis).

We compared the learning dynamics and outcome starting from two different initial positions (home postures *H*_*1*_ and *H*_*2*_; Fig 1B, top). These home postures reach to the same target in goal space but are located in different parts of the motor space (Fig 1B, top). In particular, in *H*_*1*_ the reach-endpoint is located on top of the *q*_*2*_ DoF, which modulates the effectiveness of this joint. The different home postures predict substantial differences in learning dynamics and outcome for predictions P2 and P3. If human learning is sensitive to the home posture in the ways predicted, this would indicate that also humans use local, goal-related feedback to structure exploration behaviour right from the start. The Goal Babbling simulations results are presented alongside the results from the human experiment in Sect Results for illustrative purposes.

## Results

All variables reported in this manuscript are available in the S1 Dataset.

### Performance

**Fig 2:**
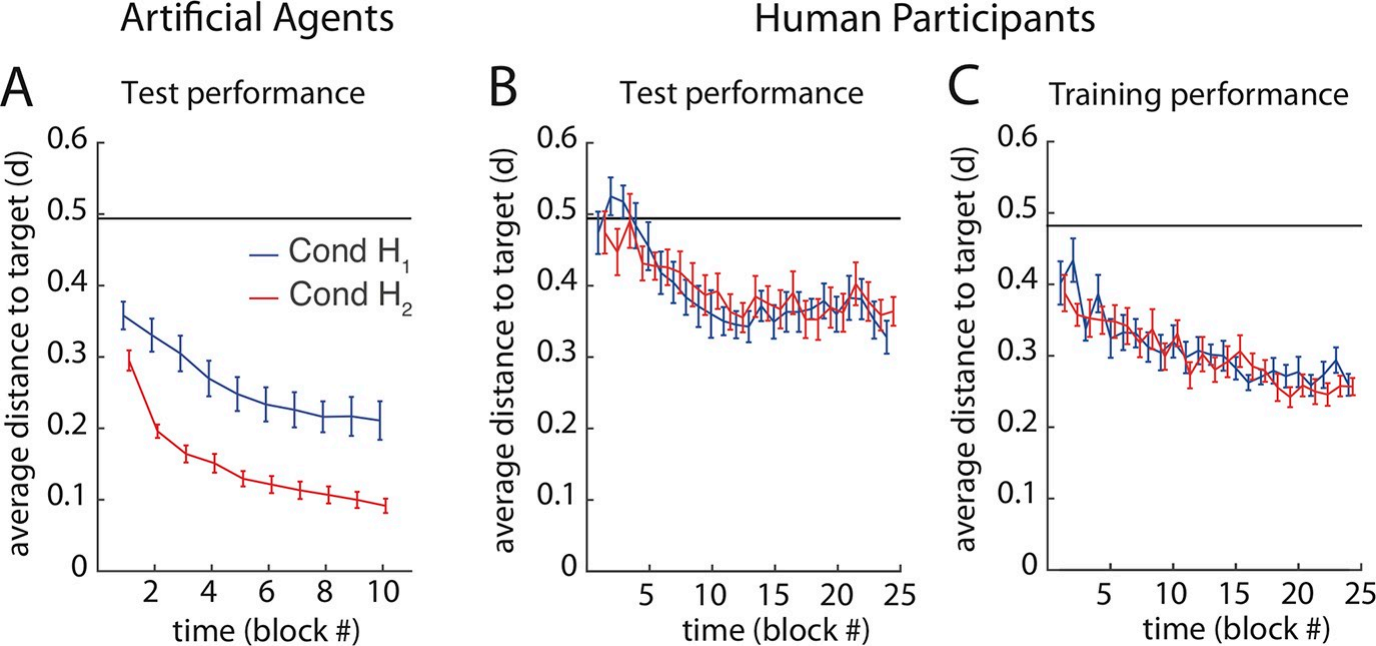
Average performance across blocks. Mean and standard error of the mean in Condition *H*_*2*_ (red) and in Condition *H*_*1*_ (blue). The black line indicates baseline performance if the hand is not moved at all (slightly lower during training as targets are spaced differently). **A:** Artificial Agents, test **B:** Human participants, test. **C:** Human participants, training.

Participants learned to perform the task in both conditions within the time allocated (70 minutes). They acquired an inverse model of the trained mapping over the course of the 24 training blocks, which allowed them to move the reach-endpoints on average closer to the target than at the beginning of training and above chance in the test blocks without feedback (Fig 2B). A two-way repeated-measures ANOVA (rmANOVA) on the average distance to the targets (error) with factors Time (block number) and Condition (*H*_*1*_, *H*_*2*_) shows a main effect of the factor Time (F(23,940) = 10.9, p≪0.001), confirming that participants learned across blocks. There are neither a significant main effect of the factor Condition (F(1,959) = 0.01, p = 0.944) nor an interaction between the factors Condition and Time (F(23,959) = 0.5, p = 0.507; full ANOVA table in S1 Table). We had hypothesised that participants might learn faster starting in home posture *H*_*2*_, as it was the case for the Artificial agents (Fig 2A). For the agents, the extended *q*_*3*_ joint in *H*_*2*_ (Fig 1B) made it easier to expand the goal space (cf. Results Sect Relative use of DoFs in synergies), so even after one block of learning, the reach error was much lower than in condition *H*_*1*_. However, there was no such condition-dependent difference in learning speed in the human population (Fig 2B and 2C).

Moreover, the human participants’ performance error remained at a higher level than in the Goal Babbling simulations (Fig 2A). Also, the test performance error (Fig 2B) converged at a higher value earlier than the training error (Fig 2C). This was likely due to proprioceptive and motor noise when executing the intended actions. More detailed results about reach performance during test can be found in S1 Fig (individual learning curves for participants), S2 Fig and S3 Fig (Distribution of reach-endpoints for individual test targets in agents (S2 Fig) and participants (S3 Fig)) and in S4 Fig (performance across time for different targets).

### Redundancy resolution

#### Motor synergy formation

To identify *Motor Synergies*, i.e., patterns of DoF co-activation that characterize a solution to the task and reduce dimensionality (prediction P1), we performed a PCA on the set of postures used to reach the targets in each three consecutive blocks (Method Sect Postures in Motor Space (q_1_, q_2_, q_3_)). Even though we were interested in inverse model learning, i.e., motor behaviour in the absence of goal-related feedback, we present results from both the training blocks (with feedback) and the test blocks (without feedback), as the training results are less noisy (cf. Fig 2B and 2C) but show the same qualitative behaviour.

**Fig 3:**
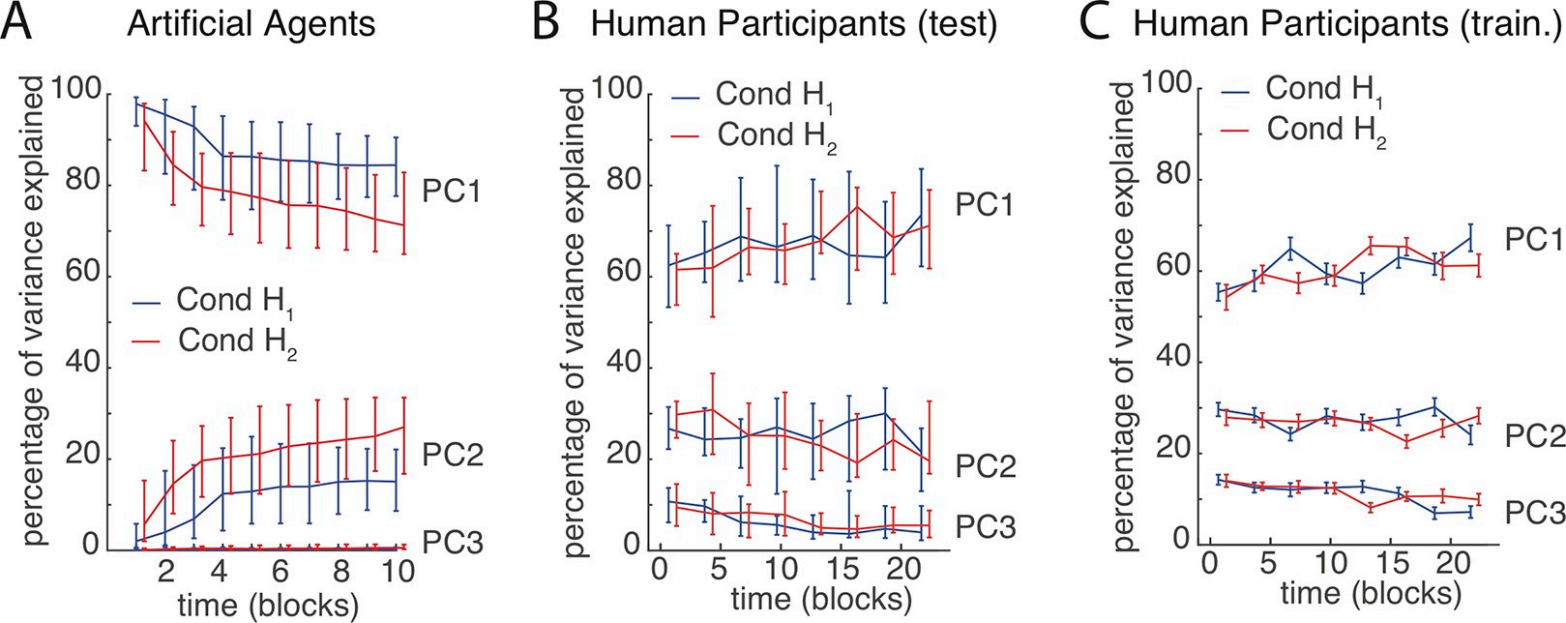
Synergy formation during motor skill acquisition. The percentage of total variance explained by each PC in artificial agents (**A**) and in human participants during the test blocks (**B**) and the training blocks (**C**). Both agents and human participants need two PCs to explain >90% of the total variance in reach postures (*q*_*1*_, *q*_*2*_, *q*_*3*_) at the end of training. In human participants, this involves a dimensionality reduction, i.e., the amount of variance explained by PC1 goes up with training (sign-test difference beginning vs. end: p = 0.041 in test; training p = 0.263), whereas the amount of variance explained by PC3 goes down (sign test difference beginning vs. end: p<0.001 in test, p = 0.041 in training). The lines in panels **A**, **B** and **C** depict the across-participant median, the error bars the interquartile range.

Fig 3A depicts the amount of variance explained by the first, second, and third principal components (motor synergies PC1, PC2 and PC3) across the artificial agent populations for both conditions. Fig 3B and 3C depicts the same measure for the human participants in the test phase (Fig 3B) and the training phase (Fig 3C). For both populations, the first two synergies PC1 and PC2 can together explain >90% of the total variance at the end of training, which is evidence for motor organization and dimensionality reduction by synergies.

For the human participants, motor organization is already evident after one block, but also increases during training: The median variance explained by PC1 increases with training blocks, whereas the variance explained by PC3 decreases (see caption Fig 3 for statistical test results; the increase in variance explained by PC1 is only significant for the test results, not the training results). In the Goal Babbling simulations, by contrast, motor behaviour is most organized right at the start: agents started off just one synergy (PC1) and only add a second movement component (PC2) over the course of learning (Fig 3A). This difference between artificial agents and human participants will be addressed again in Results Sect Relative use of DoFs in synergies and in Sect Discussion. In both populations, the formation of synergies proceeded gradually, as it would be expected from a Goal Babbling-like integrated process of learning, exploration and behaviour (P1). A transition from an exploration phase to a goal-oriented action phase, as it would be predicted by Motor Babbling, is not evident.

#### Examples of redundancy resolution

**Fig 4:**
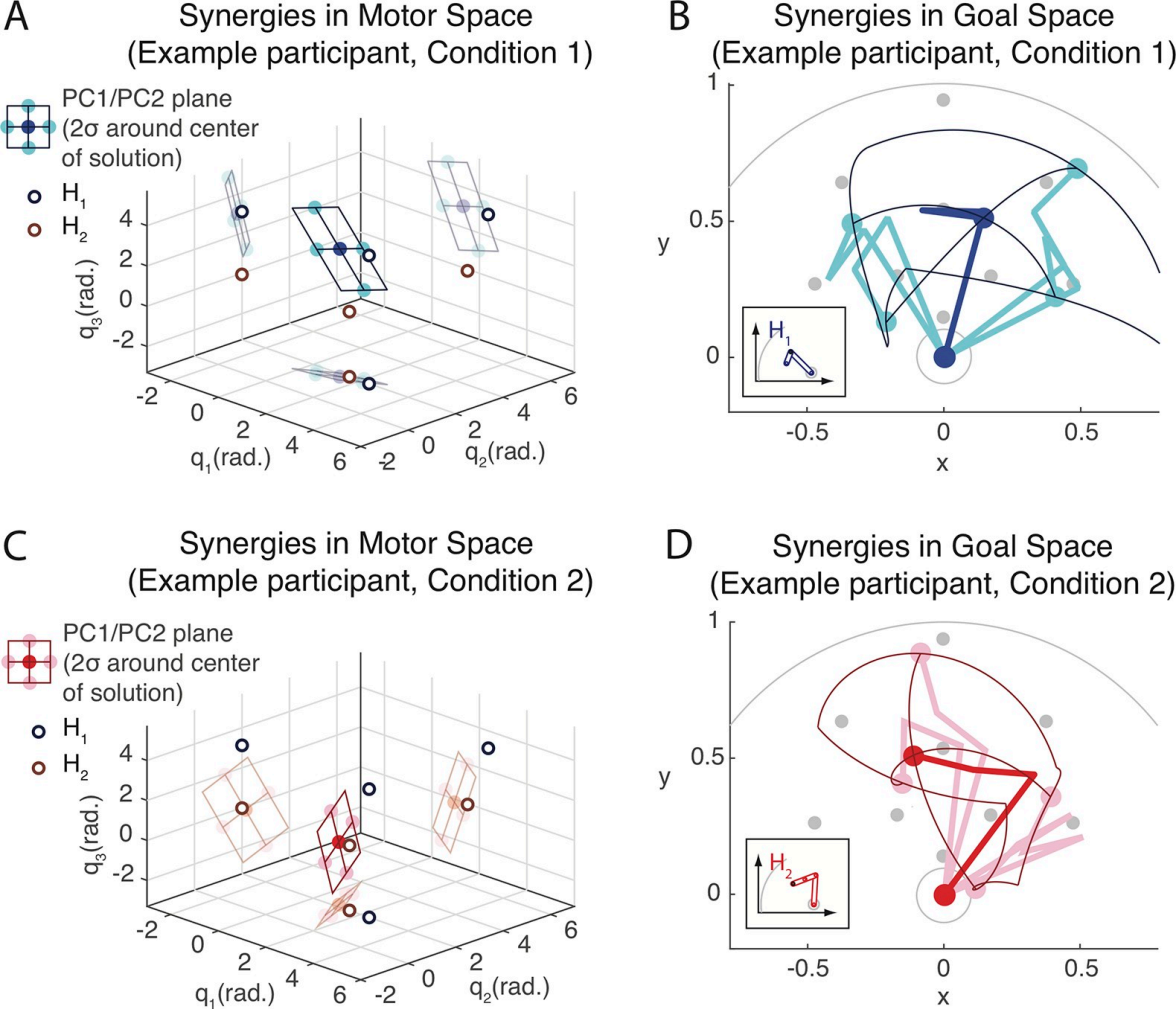
Examples of synergies learned. Two example solutions from different participants in the test phase for Condition *H*_*1*_ (**A+B**) and Condition *H*_*2*_ (**C+D**) respectively (first two synergies PC1 and PC2). Panels **A** and **C** depict the PC1/PC2 solution plane in the three-dimensional motor space. The plane extends 2σ (standard deviations) away from the central posture in the directions of PC1 and PC2 respectively. The pale ‘shadows’ are projections of the results onto the planes defined by the coordinate axes for a better impression of 3D shape. The empty disks depict the two home postures *H*_*1*_ and *H*_*2*_. Panels **B** and **D** depict the same result in the goal space (colour code is identical to **A** and **C**). The thick lines depict the arm configurations that correspond to the points of the same colour in motor space. The thin lines depict the reach-endpoints along the grid that connects the dots. The inlays depict the respective home postures *H*_*1*_ and *H*_*2*_.

Fig 4 illustrates the *motor redundancy* in the task and its solutions with two different learning outcomes for example participants tested in Condition *H*_*1*_ (Fig 4A and 4B) and Condition *H*_*2*_ (Fig 4C and 4D). These examples are not chosen because they are particularly representative, but to illustrate the across-participant variation and how they are captured by the measures investigated in Results Sects Location of solutions, Relative use of DoFs in synergies and Absolute variability.

Fig 4A and 4C show the plane defined by the first two synergies PC1 and PC2 in motor space; Fig 4B and 4D show the corresponding postures and reach-endpoints in goal space. The two solutions differ in many aspects. They occupy different regions in motor space, which means they make use of different postures to reach to the same targets, in this case postures that are more similar to their respective training home posture (cf. Fig 1B). The solutions also have different orientations in motor space: For instance, the solution learned in Condition *H*_*2*_ (Fig 4C) makes use of all three DoFs *q*_*1*_, *q*_*2*_ and *q*_*3*_, whereas the solution learned in Condition *H*_*1*_ (Fig 4A) nearly exclusively uses the *q*_*1*_ and *q*_*3*_ DoFs (different slants of the two planes). Lastly, the PC1/PC2-planes for the two solutions differ in area size: The range of joint angles used is larger in Condition *H*_*1*_ (Fig 4A) than in Condition *H*_*2*_ (Fig 4C). Despite all these differences, the two example solutions are equally viable solutions to the reaching task trained. In both cases, the reach actions associated with the solution planes cover most but not all of the area in the goal space where the test targets are located (Fig 4B and 4D).

The following sections take a closer look at the location of solutions in motor space in the light of prediction P2 (Results Sect Location of solutions), the relative use of DoFs in synergies in the light of prediction P3 (Results Sect Relative use of DoFs in synergies), and at the overall variability of postures used (Results Sect Absolute variability).

#### Location of solutions

**Fig 5:**
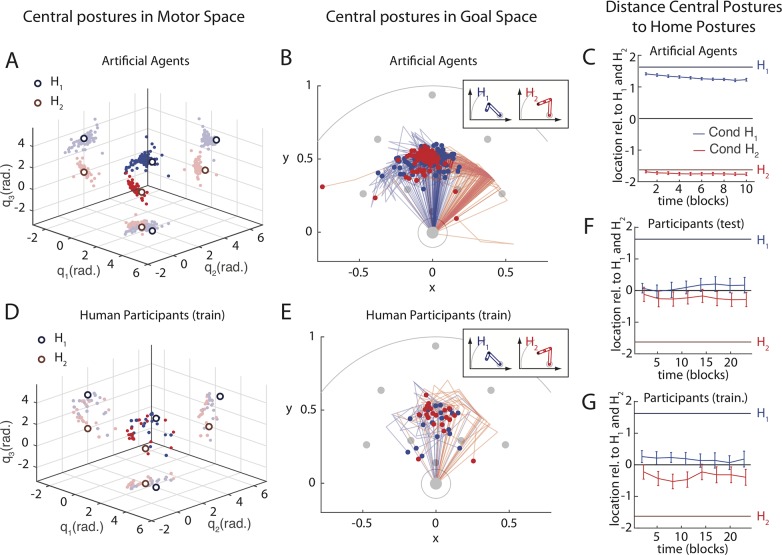
Location of learned solutions in motor and goal space at the end of training. **A-C**: Artificial Agents. **D-G**: Human Participants. Panels **A** and **D** depict the central postures (mid-point of learned solutions) in motor space for all 100 agents and 20 participants (Cond *H*_*1*_: blue, Cond *H*_*2*_: red). Panels **B** and **E** depict the corresponding arm configurations in goal space, which shows that the reach-endpoints of the central postures cluster around the mid-point of the array of test targets. Panels **C**, **F** and **G** depict the average location of solutions relative to *H*_*1*_ and *H*_*2*_ across time (population mean and standard error of the projection on the line connecting *H*_*1*_ and *H*_*2*_). Both agents (**C)** and humans (test **F**, training **G**) are biased towards the starting home posture, but this bias is weaker in humans.

Fig 5A and 5D show the postures at the centre of the learned solution manifolds (central postures) for artificial agents (Fig 5A) and for human participants (Fig 5D) at the end of training. In the agent population, the distribution of learned central postures follows an arch in motor space that connects *H*_*1*_ and *H*_*2*_ (Fig 5A). This arch corresponds to a manifold of postures that reach to the central target in goal space (Fig 5B). The central postures remain very close to the training home posture in motor space (Fig 5C). This is owed to the Goal Babbling learning procedure, which introduces small variations to gradually expand the motor repertoire from the home posture in the direction of behavioural success (prediction P2). In Motor Babbling, no such influence of the home posture is expected, as the random initial exploration is exhaustive, not local, which should lead to identical learning outcomes (forward and inverse models) in both conditions.

The human test population showed the same pattern as the agents who learn the task by Goal Babbling, albeit with more noise and a weaker bias towards the home posture (Fig 5D–5G): A two-way rmANOVA with the factors Condition and Time reveals a significant main effect of the factor Condition (F(1,300) = 4.6, p = 0.046), i.e., the learned solutions are biased towards the home posture. There is no main effect of the factor Time (F(7,300) = 0.4, p = 0.875) and no interaction between the factors Time and Condition (F(7,300) = 0.6, p = 0.725; full ANOVA table in S2 Table).

#### Relative use of DoFs in synergies

**Fig 6:**
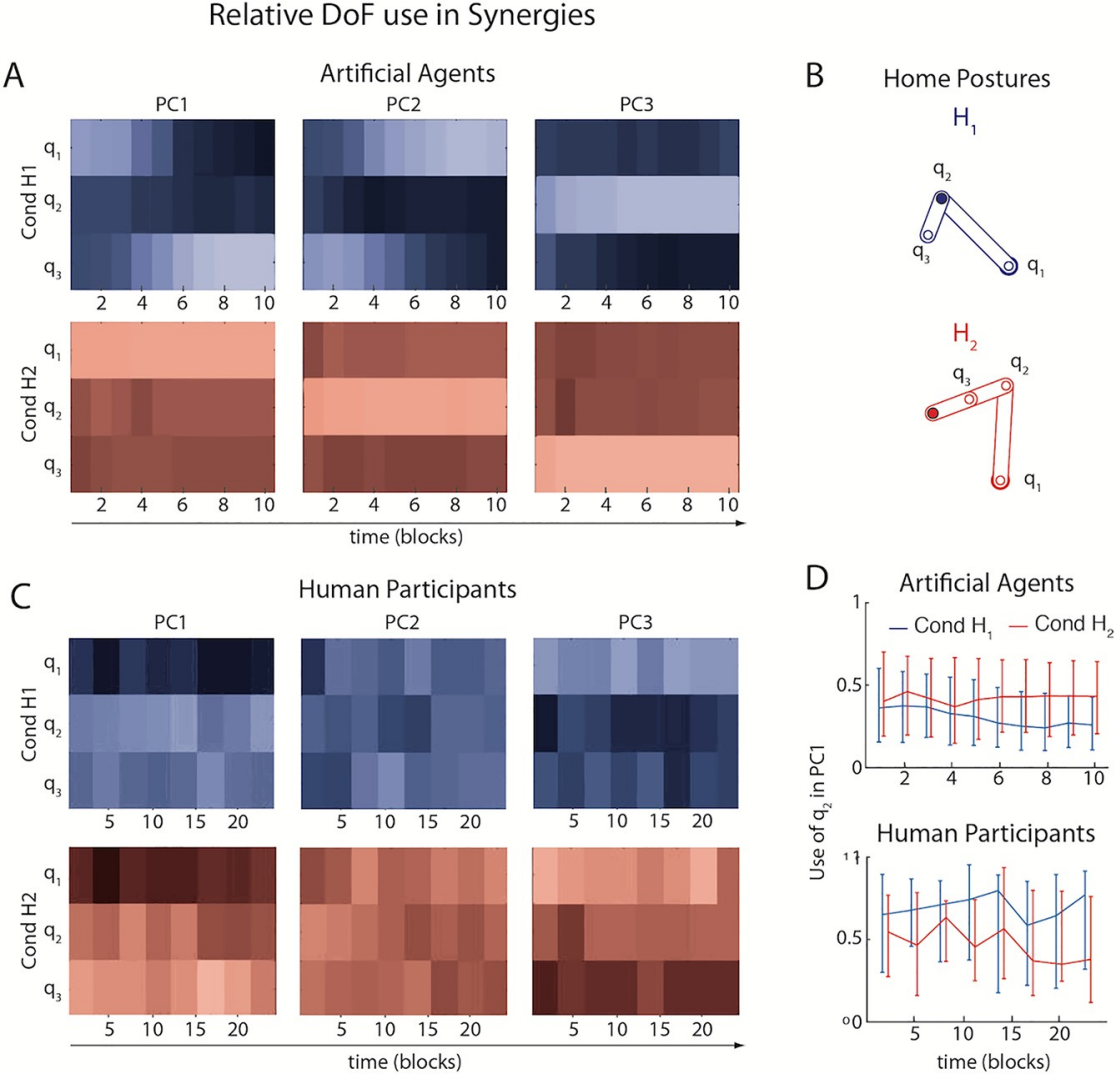
The relative use of DoFs in learned synergies. **A+C:** Population median of the absolute values of *q*_*1*_, *q*_*2*_ and *q*_*3*_ in PC1, PC2 and PC3 (unit length vectors) in artificial agents (**A**) and human participants during training (**C**). Brighter colours indicate a higher absolute value of the respective DoF. **B:** Illustration of the home postures *H*_*1*_ (blue) and *H*_*2*_ (red) in goal space. Note that the *q*_*3*_ joint is contracted in *H*_*1*_ and extended in *H*_*2*_. **D:** Median and interquartile range of absolute *q*_*2*_ values in PC1 across agents (top) and participants during training (bottom).

To assess the relative use of DoFs, we analysed the absolute *q*_*1*_, *q*_*2*_ and *q*_*3*_ components in the first two synergies, and in particular the use of *q*_*2*_ in the first synergy (PC1). In Goal Babbling, motor learning favours DoFs that cause maximum variability in the goal space, as the exploratory noise has the biggest task-error-reducing effect when using these DoFs (prediction P3; [cf.^30^]). We selected the training home postures with this constraint in mind. In *H*_*1*_, the *q*_*3*_ DoF is fully contracted (cf. Fig 6B), which renders movement of the *q*_*2*_ DoF ineffective in goal space, as the reach-endpoint rests on top of the *q*_*2*_ joint. Therefore, the *q*_*2*_ DoF did not play a prominent role in the solution learned by the agents in Condition *H*_*1*_ (Fig 6A top). By contrast, in *H*_*2*_ the *q*_*3*_ joint is fully extended. Both configurations are usually singular in actual robotics but this is not important for the purposes of the experiment presented here. This makes movement of the *q*_*2*_ DoF maximally effective in the goal space, so the agents incorporated *q*_*2*_ movements into the learned solution (Fig 6A bottom). This difference increased with training (Fig 6A and 6D top). In agreement with previous work [30], variance in the DoFs that have the smallest effect on reach-endpoints (*q*_*2*_ in Condition *H*_*1*_, *q*_*3*_ in Condition *H*_*2*_) is mostly explained by PC3, which contributes least to solving the task.

The results from human participants differed substantially from the Goal Babbling simulation results and are overall noisier. However, it almost appears as if PC1 and PC3 were inverted between the two populations (cf. Fig 6A and 6C). In both conditions, *q*_*1*_ contributed least to PC1 in the human participants. In both conditions, the variance of *q*_*1*_ is largely explained by PC3, that is the least relevant movement component. Also, the influence of home posture on the use of *q*_*2*_ in PC1 follows the opposite trend for the human participants: The *q*_*2*_ DoF was used more in Condition *H*_*1*_, where it was ineffective, than in Condition *H*_*2*_ (Fig 6C and 6D bottom). However, this trend is not significant (Wilcoxon signed-rank test of the median difference between conditions across time: z = 1.38, p = 0.167). The initial exploratory activity was not as strongly modulated by the local effectiveness of DoFs as in the simulated agents, and any possible trend has the opposite polarity: Increased local effectiveness of a DoF tended to diminish the use of DoFs instead of boosting it.

This trend to invert prediction P3 is neither agreeable with the Goal Babbling simulation nor with a Motor Babbling explanation, which would predict an equal use of all DoFs during the exploratory phase, irrespective of how effective they are. This pattern suggests that goal-relevant feedback might have been used in a different way by the human participants than by the Goal Babbling agents (see Discussion). However, as this trend is not significant, further experiment with less motor noise would be required to draw conclusions from this.

#### Absolute variability

**Fig 7:**
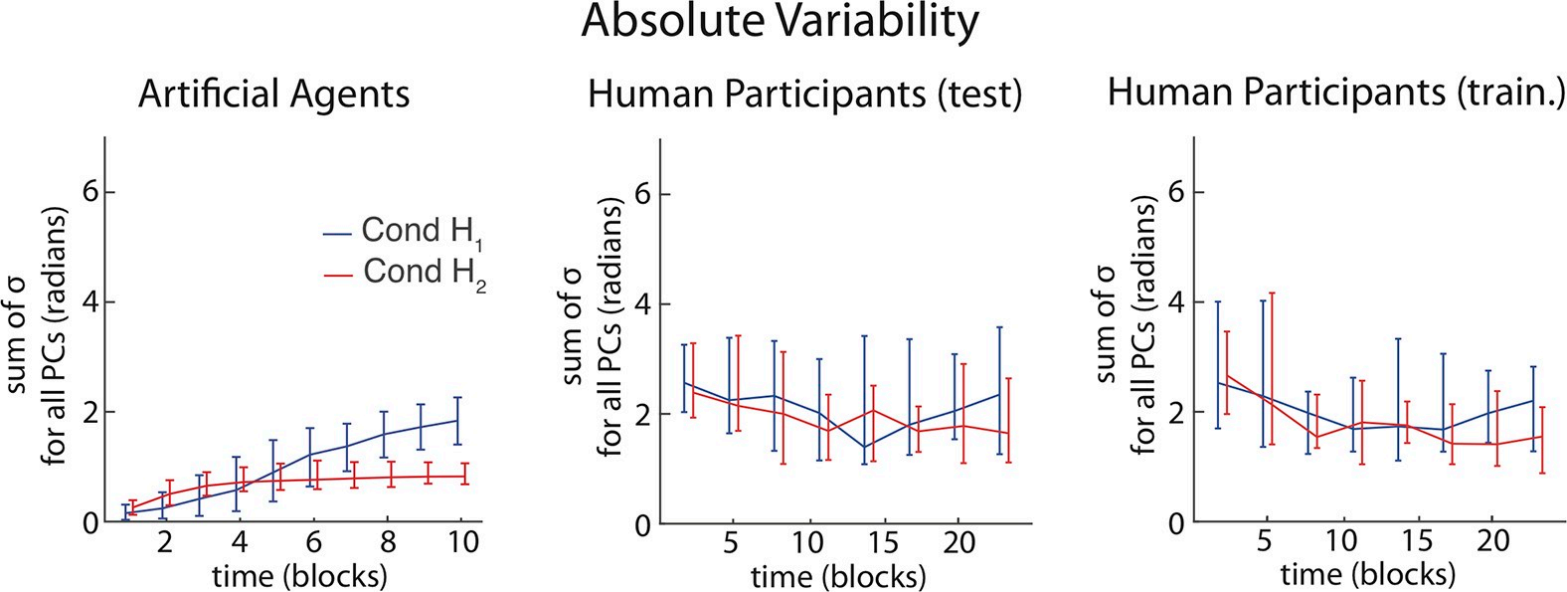
Absolute variability and influence of finger mapping on motor organization. The summed variance of all three synergies for artificial agents (left) and human participants (right) across time (median and interquartile range).

In Goal Babbling, local noise around a home posture leads to a gradual, feedback-guided expansion of the region in motor space that is used to solve a task (Sect Goal Babbling Simulations). This can be seen in the summed variance in motor space (Fig 7 left), which starts at zero in the artificial agent population, grows during learning and converges when a functional solution is found. In humans, overall variability started high and decreased across time (a two-way ANOVA with the factors Time and Condition shows a main effect of the factor Time: F(7,300) = 2.4, p = 0.024), which again is an inversion of the behaviour observed for agents. The overall variability in postures used was not modulated by the home posture (no main effect of factor: Condition; F(1,300) = 0.1, p = 0.762 and no interaction between the factors Time and Condition; F(7,300) = 1.0, p = 0.457; full ANOVA table in S3 Table).

This difference between agents and humans might explain some of the previously reported differences between the two populations. It appears that humans initially explored large parts of the motor space (high variability, Fig 7 right) to sample it with comparably little direction or structure (more synergies are necessary to explain initial behaviour, Fig 3B and 3C). In this sense, human exploration can be seen as more random and less goal-directed than in the population of Goal Babbling agents. However, the evidence for synergy formation (P1, Sect Motor Synergy Formation) and a location bias (P2, Results Sect Location of solutions) starting from the first block of experimentation onwards and throughout the experiment still is evidence for a Goal Babbling-like integrated exploration, learning and goal-oriented action process (with a large learning rate or a lot of exploratory noise) in humans and cannot be explained by Motor Babbling, which predicts a random exploration phase followed by a goal-directed action phase.

Still, this higher randomness could explain why humans have avoided the use of DoFs with extreme consequences in the goal space, as random and large exploratory movements can lead to unstable effects in the task. This could then also explain why the first two synergies in the human population do not involve the *q*_*1*_ DoF, which has a high local effectiveness (inversion of prediction P3, Results Sect Relative use of DoFs in synergies). The artificial agents by contrast expanded their motor repertoire only in very small steps (Fig 7 left), so they had no behavioural instability and thus initially favoured DoFs with a strong local effectiveness that allowed introducing more variance in reach-endpoints (Fig 6A and 6D). The near exclusive use of the *q*_*1*_ DoF, which is the most effective DoF, can initially explain nearly all of the variance in postures in the simulated agents (Fig 3A).

In this interpretation, both humans and artificial agents used goal-related feedback to guide motor exploration and redundancy resolution, which is why systematic differences in the use of DoFs occurred in both populations (Fig 6). However, the motor exploratory strategies differed dramatically between the two populations (Fig 7), which could explain why the differential use of DoFs follows the opposite pattern in agents and humans (Fig 6A vs. Fig 6C) and why the effect of home posture is weaker in the human population (Fig 5F and 5G, Fig 6C and 6D). There are a number of possible reasons for why humans might have used a different exploratory strategy than the agents in the given task (cf. Sect Discussion).

### Human morphology and task motor space

Motor skill acquisition in human adults involves adding a new layer of motor control on top of the already existing control of the body in space. In the current experiment, existing motor synergies for finger movements and biomechanical constraints of the hand likely had an effect on motor skill acquisition. To decrease these effects, we chose to randomize the assignment of fingers to task DoFs and counter-balanced this assignment across conditions.

**Fig 8:**
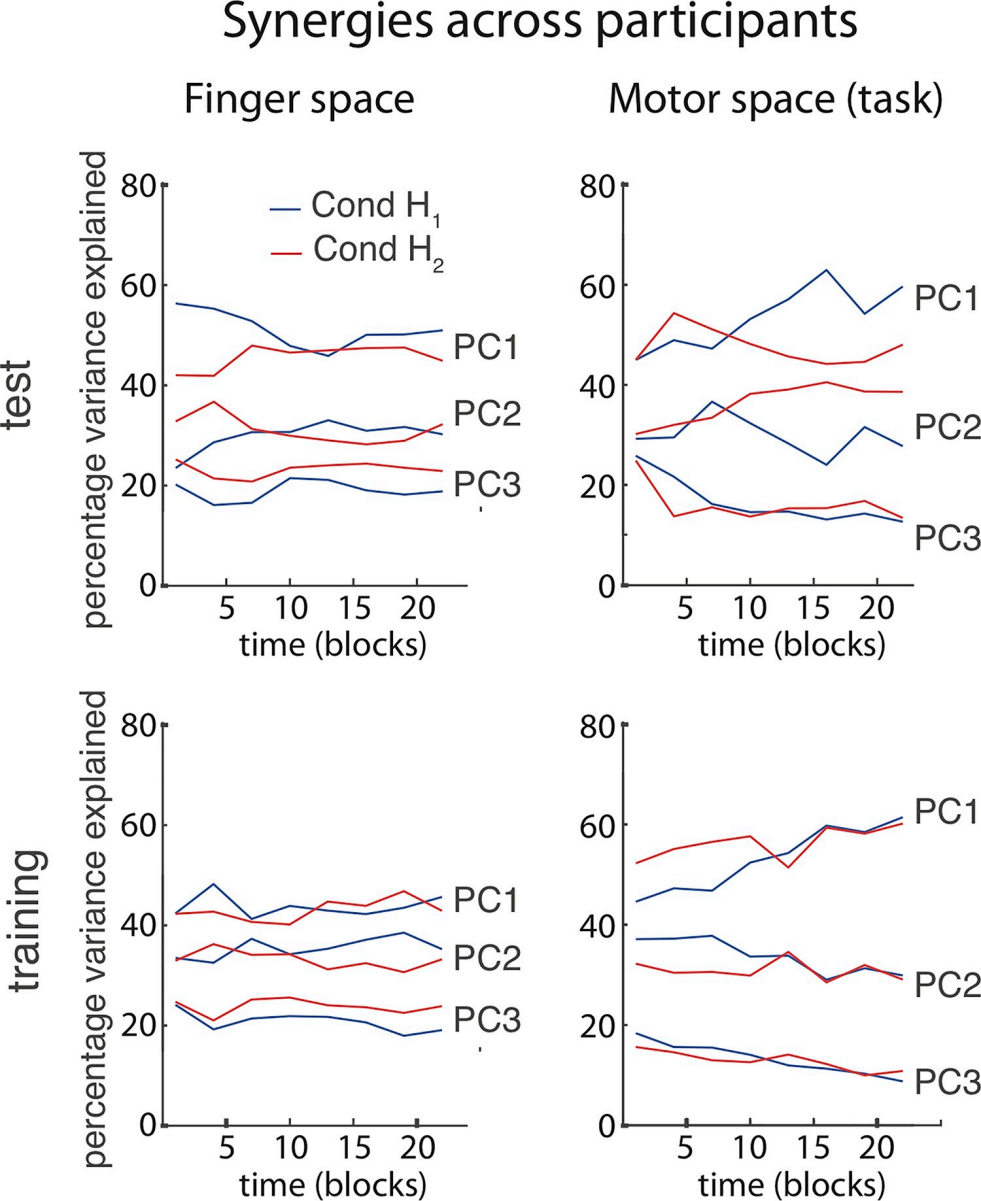
Percentage of variance in the across-participant reach-endpoint space that is explained by the first, second and third PC across time. More variance explained by the first synergies indicates more organization of motor control. Left: PCA on human morphology (left index, right index, right middle fingers). Right: PCA on the task motor space (*q*_*1*_, *q*_*2*_, *q*_*3*_). Top: test blocks, bottom: training blocks.

To get an idea of the strength of such biasing effects of human morphology, we performed two additional PCAs on the reach-endpoints pooled across participants, to compare the amount of motor organization in the biological morphology motor space with the amount of motor organization in the task motor space. That is, in a first PCA, we looked for motor synergies for physical finger control (human morphology) and in a second PCA, we looked for motor synergies for task DoF control (*q*_*1*_, *q*_*2*_ and *q*_*3*_). More variance explained by the first synergies (PCs) indicates more organization of motor control. Fig 8 shows the variance explained by PC1, PC2 and PC3 in both analyses.

In all test blocks, the first two synergies of finger movement (PC1 and PC2, human morphology) can explain approximately 80% of the overall variance throughout the experiment (Fig 8, left). This confirms that there was above-chance motor organization in finger control, i.e., participants used patterns of finger co-activation. Thus, human morphology influenced motor organization throughout the experiment.

In the task motor space, the variance explained by the first two synergies (PC1 and PC2) started at a similar level but then increased across the learning phase (Fig 8, right), to the point that the first two synergies explain 90% of the variance at the end of the experiment. The learned motor organization in task motor space is stronger than the organization in the previously existing morphological motor space.

## Discussion

We here investigated whether humans use goal-related feedback to explore an unfamiliar, redundant, and non-linear visuomotor mapping and whether they use such feedback to constrain the inverse model from goal states to motor states right from the start of exposure. Little is known to date about the dynamics of inverse model learning in human motor control and in robotics. To this end, we tested whether initial state (home posture) influences human behaviour in ways predicted by the Goal Babbling learning rule, an inverse model learning rule for robotics that works even for high-dimensional robotic platforms.

We observed that learned solutions rely on the use of motor synergies from the first block onwards that keep being refined throughout the experiment (prediction P1, Results Sect Motor Synergy Formation) and that the solutions are biased to remain close to the training home posture in motor space (prediction P2, Results Sect Location of solutions). There is also a trend for the home posture to bias exploratory behaviour (prediction P3, Results Sect Relative use of DoFs in synergies): DoFs that are locally less effective appear to be initially favoured, which suggests that goal-related feedback structures exploration behaviour right from the start, but in different ways than predicted. Altogether, these results show that motor exploration is guided by goal-related local feedback throughout the experiment, as is the case in the Goal Babbling learning rule, not random in an initial exploration phase and structured in a later goal-oriented action phase, as Motor Babbling models assume [12,13].

Despite these similarities between human behaviour and Goal Babbling simulations, there are also important qualitative differences between simulated agents and human participants. In the Goal Babbling simulations, small variations around the home posture cause a gradual expansion of the motor repertoire away from the home posture into the direction of behavioural success. This gradual expansion is strongly structured by goal-related feedback. By contrast, the human participants in our study initially explored the space by performing large (Fig 7) and less structured (Fig 3) movements, that is, their exploration was more random than in the agent population.

It is important to note that, though more random than in the agent, exploration was still more structured than would be expected of random Motor Babbling: even after one block of experimentation (80 s), movements were already organized in synergies (P1) and were confined in motor space (P2). There is no evidence for a transition from a random exploration phase to a structured behaviour phase, as predicted by Motor Babbling models, even though performance was still poor and learning continued for another 70 minutes. Therefore, the randomness in human behaviour can be better described as exploratory noise with a large magnitude than as Motor Babbling.

It is however theoretically possible that Motor Babbling behaviour might have occurred within the first block (80s); our experiment was not designed to identify learning processes that might take place in a sub-minute time scale. Even if this were the case, learning that was guided by goal-directed feedback was clearly the dominant process over the course of the experiment.

This experiment shows that direct inverse model learning can happen also when adult humans learn new unfamiliar and redundant sensorimotor mappings. From this result, we cannot rule out that humans might learn new sensorimotor mappings in a more phase-wise manner in different scenarios. We also did not explicitly seek a comparison with other theories or computational models of motor acquisition that have no predictions for inverse model learning. For instance, some of our results might also be explained by a nearest-neighbour reinforcement learning strategy, though the continuous nature of the task and the fact that participants and robots comfortably generalize also to targets outside the learned range of targets (See S4 Fig) speaks against that possibility. There are still a lot of open questions about how humans learn new and redundant sensorimotor mappings and we hope that our result and paradigm give researchers a new angle on this problem.

One question that remains is why participants had a more random initial exploration strategy. A possible explanation is that they had to dynamically infer a suitable learning rate in a way that the agents did not. That is, the initially large exploratory movements might have served the purpose to estimate a reasonable range of finger movements in this task. The agents in the chosen implementation of Goal Babbling were bound to a fixed, task-specific learning rate that was set by their engineer to be low enough to avoid destabilization of control yet high enough for learning to converge in a reasonable amount of time. The freedom to autonomously derive and adapt the learning rate gives the human participants more autonomy to successfully learn in a larger range of learning scenarios compared to the chosen implementation of Goal Babbling. Attempts to include such active learning behaviour also in robotics have recently been made, for instance, in forward (Motor Babbling) exploration [37] and in Goal Babbling [38], where the motor space can be explored through so-called direction sampling in a larger-scale manner without explicitly setting the goals beforehand.

Another possible explanation for the differences in motor exploratory strategy is that the motor task given to the human participants is not in all aspects a typical example of human inverse modelling. For instance, it does not harbour any danger of injury, pain or behavioural breakdown, which makes a conservative learning rate unnecessary and encourages a ‘riskier’ exploratory strategy. In many situations where humans acquire new motor skills and specifically during infant development, large exploratory movements might be more dangerous or injury-prone and a more careful motor exploration might be performed.

Independent of their origin, the differences in exploratory strategy might explain why DoF-effectiveness has the opposite effect on humans as it does on simulated agents for motor synergy learning (prediction P3, Results Sect Relative use of DoFs in synergies). In an effort to stabilize an unstable control system, it would make sense for humans to attenuate DoFs whose movements have drastic sensory consequences and instead rely more on DoFs whose movements are less affected by motor noise. Agents learning an inverse model by Goal Babbling instead are eager to ‘escape’ the narrow range of target reaches they are initially able to perform and learn according to a cost function that minimizes effort (Method Sect Goal Babbling Simulations). Goal Babbling thus favours more effective DoFs and DoFs with more noise [30] (see also [39] for a similar trend in human behaviour in a different motor learning task).

Overall, the magnitude of differences between conditions in the human population were small compared to the Goal Babbling simulations. Again, the noisier exploratory strategy is a possible reason for this: If the local search spaces are larger for humans, they are more likely to overlap, especially given that the task used is not really high-dimensional, compared to other tasks used for the study of redundancy resolution in human motor learning [28,35, 40, 41]. In our task, an exhaustive sampling of the three DoF motor space, as proposed in Motor Babbling, would still be possible. Our rationale for keeping the motor space comparably low-dimensional is that it allowed us to analyse the role of specific DoFs in motor skill acquisition in an intelligible manner that is easy to interpret. Moreover, previous studies on learning unfamiliar sensorimotor mappings used existing motor synergies as building blocks for learning, which we tried to avoid by randomizing the assignment between fingers and task DoFs across participants.

Another possible source of differences between agent and human participant behaviour lies in differences in the task. For instance, in the setup, there is the baseline posture q* (Methods Sect Setup, Fig 7B left) as an across-condition salient posture. The baseline posture is achieved when participants hold their fingers level on the visual reference line and which is located between the two home postures *H*_*1*_ and *H*_*2*_ in motor space. It is well possible that this baseline posture attracts motor exploration in both conditions. This might have diminished the effect of home posture in the human population, especially as keeping the fingers in the baseline posture also corresponds to a comfortable hand posture for the participants (fingers kept level in the plane of the hand mount). Also, the visual display of finger position feedback throughout the experiment might have emphasised the role of visual feedback in ways not usually encountered in motor skill acquisition. There are many factors that distinguish the experiment with human participants from the agent simulations, so the comparative analysis should not be overstrained. We were mostly interested in a qualitative comparison of the two types of system, whether their learning can be structured into phases of exploration and goal-directed action and how initial state and goal-related feedback influence the solutions learned.

Little is known to date about how humans explore unfamiliar motor spaces and how they constrain redundant sensorimotor mappings. Motor Babbling approaches struggle to explain how inverse models might be learned in high-dimensional and redundant systems, where a random and exhaustive exploration phase is not feasible. Goal Babbling exploits goal-related feedback for motor exploration and learning and thus makes inverse model learning in high-dimensional platforms possible. We could show that, in the present experiment, like in Goal Babbling, participants use local goal-related feedback to guide exploratory behaviour and resolve motor redundancy right from the start of the experiment. Even if the exploratory behaviour differs between artificial agents and human participants, who use a larger and less structured initial range of movements, the learning in both cases can be best explained as an integrated process, where goal-oriented action and inverse model learning mutually bootstrap and bias one another, not as a stage-wise process where the sensorimotor mappings are learned first and then put to use in a second, distinct phase of learning and goal-oriented action.

## Methods

### Ethics statement

The experiment was approved by the ethics committee of the Bielefeld University and was conducted according to the principles expressed in the Declaration of Helsinki. Informed written consent was obtained from the participants.

### Task

Both humans and simulated agents were trained in the same planar reaching task with a three DoF planar arm (Fig 1A). The three segments *l*_*1*_, *l*_*2*_ and *l*_*3*_ of the arm are of length *l*_*1*_ = 0.55, *l*_*2*_ = 0.225 and *l*_*3*_ = 0.225 (length in arbitrary spatial units). No joint limits were implemented: The joint angles that define a posture of the arm (*q*_*1*_, *q*_*2*_, *q*_*3*_) could be set to any value in [0, 2π [(angles in radians).

Two different home postures *H*_*1*_ and *H*_*2*_ were defined as starting conditions for learning (Fig 1B; *H*_*1*_ = (¾ π, 1.99, π), blue; *H*_*2*_ = (1.51, 1.99, 0), red). These home postures reach to the same point (-0.39, 0.39) in goal space but are located in different parts of the motor space (Fig 1B).

The home postures are located at the edge of an array of 16 training targets that are distributed in a regular manner (in polar coordinates) in a quarter segment of the arm’s reach space (Fig 1B; distances from origin: [0.25, 0.45, 0.65, 0.85]; angles: [¼ π, 5/12 π, 7/12 π, ¾ π]). Nine additional targets were used as test targets (Fig 1B); five within the training array (polar coordinates: (0.35, 2/3 π), (0.75, 2/3 π), (0.55, ½ π), (0.35, 4/3 π), (0.75, 4/3 π)) and four just outside the training array (polar coordinates: (0.55, 1/6 π), (0.15, ½ π), (0.95, ½ π), (0.55, 5/6 π)).

### Goal babbling simulations

A population of 100 simulated agents was trained on the task using the Goal Babbling procedure, which we here describe only briefly. For technical details of the procedure, please refer to [24] or to pages 65–70 of [42].

The goal space and motor space for the simulated agents were identical to those used in the experiment with human participants (Method Sect Task). That is, the forward kinematics are given by

(x,y)=f(q) (1)

where $q\in R_{3}$ are the joint angles. Goal Babbling aims at learning the inverse model:

q=g(x,y) (2)

which maps desired outcomes (hand positions) to actions (joint angle configurations) such that (*x*,*y*) ≈ *f*(*g*(*x*,*y*)) for a set of targets *X*,Y* in the goal space. Training and test targets were identical to those used in the experiment with human participants (see visualization in Fig 1).

**Fig 9:**
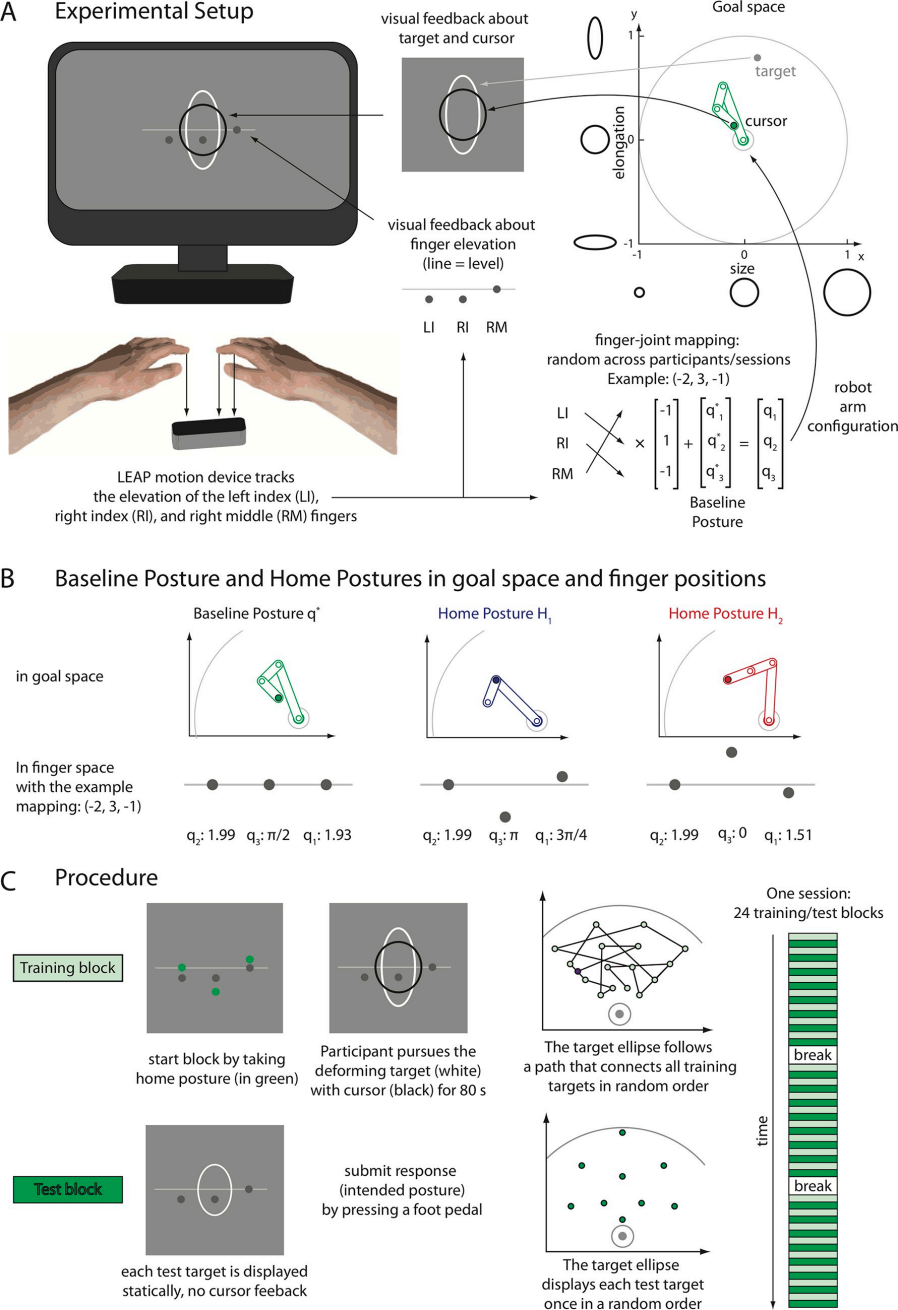
Setup and Procedure for the Experiment with Human Participants. **A:** Participants experience the task as deforming a black ellipse to track the shape of a white ellipse by moving their left index (LI), right index (RI) and right middle (RM) fingers up and down (left). Internally, finger positions are mapped to the joint angles *q*_*1*_, *q*_*2*_ and *q*_*3*_ of the 3 DoF planar arm (bottom right, Method Sect Task). The size and elongation of the ellipses represents the position of target and reach-endpoint in goal space (top right). **B:** When a participant keeps the three fingers LI, RI and MI level (bottom left), this corresponds to taking the Baseline Posture q* (top left), which is in the middle of the two home postures (top middle and top right) in motor space. To take one of the home postures, the fingers have to be moved away from the midline in a manner that depends on the random mapping between fingers and joints (example mapping: (-2, 3, -1)). **C:** Each session consisted of 24 training and test blocks (right). A training block (top left) was started by taking the home posture. The deforming white target ellipse had to be tracked with the black ellipse (reach-endpoint) continuously for the next 80 s. In test blocks, only the white target ellipse was displayed and participants moved their finger till they reached a configuration they thought corresponds to the target and then submitted their response by pressing a foot pedal.

A local linear map with learning rate *η* = 0.2 is used [42,43] as learning model for the inverse function. Training starts at the home posture *H*_*1*_ or *H*_*2*_ (cf. Method Sect Task), depending on condition. The agent is trained using the training targets (see Fig 9C). Training involved moving the hand from a given training target x,y*(*t*_*n*_) to the next training target x,y*(*t*_*n+1*_) in 25 steps. That is, 24 sub-targets were generated to transition from the current training target x,y*(*t*_*n*_) to the next training target x,y*(*t*_*n+1*_) by linear interpolation in the goal space. The agent tried to reach these targets by utilizing the respective current estimate of the inverse function $\hat{q}(t)$. After each step in each trial the estimate of the inverse function is immediately updated (online learning).

In each trial, exploratory noise *e* was added to the outcome of the local linear map estimate of the inverse function $\hat{q}(t)$ to generate novel behaviour and gradually expand the explored region of the motor space.

$$q^{+}(t)=\hat{q}(t)+e(x,y*(t),t)$$ (3)

The noise *e* follows a slow and bounded random walk of linear perturbations as defined in [42], p. 68 (Equations 6.8–6.10 with parameter values *σ* = 0.5,*σ*_Δ_ = 0.005). The amount of noise was equal for all joints *q*_*i*_. Implementing exploratory noise as a random walk ensures both continuity of behaviour (to avoid instability) and ensures that, over time, previously unknown parts of the motor space are explored.

A training block involved the presentation of 40 training targets that were selected at random from the set defined in Method Sect Task. The home posture was chosen as a target with probability 0.1. Considering the 24 sub-targets on the way to the next training target, a total of 1000 parameter updates were thus performed during learning in one block.

The agents were trained in ten subsequent blocks. Note that these blocks have no special meaning in the learning algorithm. They are solely distinguished for evaluation purposes, i.e., to assess the current estimate at regular intervals using the nine test targets that are also shown in Fig 9C. During these test blocks, the learning dynamics were disabled.

All simulation results show the average and variance over a population of 100 agents, which differ according to the random exploration scheme (*exploratory noise*) described above. These results are presented merely to qualitatively illustrate the kind of behaviour to be expected from Goal Babbling-like learning in the participants, as it is known from the literature and reflected in the experimental hypotheses. We therefore do not phrase or test any hypotheses about the behaviour of the simulated agents or how it quantitatively compares to the behaviour exhibited by human participants.

### Experimental setup and procedure

To avoid that participants use prior knowledge about arm kinematics, space or physics to learn the task, we applied an unintuitive and unfamiliar transformation to the DoFs in motor space (joint angles *q*_*1*_, *q*_*2*_, *q*_*3*_) and the feedback about target and reach-endpoint position (*x* and *y* coordinates). Participants controlled joint angles *q*_*i*_ by elevation of the left index, right index and right middle fingers (Fig 9A). They received visual feedback about reach-endpoint and target as deformation of two static ellipses (Fig 9A). That is, formally speaking, participants were controlling movements of a three DoF planar arm but their experience of the experiment was that of moving the fingers up and down to deform an ellipse. The assignment of fingers to DoFs of the simulated planar arm was randomized across participants and conditions (see Procedure). S1 Video shows a participant performing the task for illustration.

#### Population

A total of 20 healthy adult volunteers participated in the experiment (all self-reported to be right-handed; 10 female, age range 19–25). They received a small monetary compensation for their participation (6 Euro per hour). Upon arrival at the laboratory for their first session, they read and signed an informed consent form.

#### Setup

The experiment was implemented using the Psychophysics Toolbox for MATLAB [44]. Participants were seated in a dark room with their hands fixated in a mount 12.5 cm above a LEAP Motion sensor that recorded the movements of participants’ hands and fingers. The positions of the fingertips were read out with a custom-written C++ routine that used the API and skeletal fitting algorithm of the LEAP SDK [45] and relayed the information to the experimental program via UDP. Only the vertical position of three fingers (left index (LI), right index (RI) and right middle (RM)) was used.

Throughout the experiment, visual feedback about the elevation of the three relevant fingers was displayed on a standard LCD computer monitor (60 Hz refresh rate, 50% grey background) at approximately 60 cm viewing distance. Three 25% grey disks with a radius of 0.2° visual angle and a horizontal distance of 2.6° visual angle between disks corresponded to the positions of LI, RI and RM fingers respectively (Fig 9A). There was also a 75% grey horizontal reference line at the centre of the display, which corresponds to keeping the fingers level in the same plane as the top of the hand mount on which participants rest their wrists and forearms. Vertical finger movements of 1 cm in the real world caused a corresponding vertical dot movement of 0.69 cm on the screen. Displaying feedback about the finger position had the dual purpose to ease the generation of intended postures despite noisy proprioceptive information and to ensure appropriate recording with the LEAP sensor. If participants unconsciously rounded their hands, this led to occlusion problems and instability in the hand tracking. In such a case, the feedback dots disappeared or failed to follow the finger movements, and participants were instructed to spread their fingers if this happened.

Each finger’s vertical position is interpreted as one of the angles *q*_*1*_, *q*_*2*_ and *q*_*3*_ that together determine a reach-endpoint position in goal space (cf. *Task*). Five cm of finger movement correspond to a change of π in angle. Which finger controlled which joint was randomized (details about the assignment from fingers to joints in Method Sect Procedure). If all fingers were held level on the visual reference line (Fig 9B, left), this was interpreted as a joint configuration we call the Baseline Posture q^*^ = (1.93, 1.99, ½ π), which is in the middle between the two home postures *H*_*1*_ and *H*_*2*_ in motor space (cf. Fig 9B).

Feedback about the target position in goal space was displayed as white stationary ellipse, feedback about the reach-endpoint position as black stationary ellipse. This goal feedback was presented on top of the visual feedback about finger positions (Fig 9A, top left). The *x* coordinate determines the size of the ellipse; the *y* coordinate determines its elongation (Fig 9A, top right). The *x* coordinates in [–1,1] are linearly mapped to the base radius *r* in [0.66°, 3.30°] visual angle. The y coordinates in [–1,1] were then mapped linearly to the range [-0.5, 0.5] to determine the elongation factor *e* that distorts the circle defined by the base radius *r*. The horizontal radius was given by *r*_*horz*_ *= 1-e* and vertical radius by *r*_*vert*_ *= 1+e*.

#### Procedure

Participants were tested in two sessions of 90–120 minutes on different days (approximately 70 minutes of experimentation and an additional 20 minutes of breaks). In each session, they were tested in one condition (home posture *H*_*1*_; home posture *H*_*2*_). The starting condition was counterbalanced across participants. At the beginning of the first session, participants underwent a short (less than five minutes) training to familiarize themselves with the procedure and setup.

The assignment of fingers to joints and the direction of the mapping differed between the two sessions. For instance, a mapping of [–2, 3, –1] implies that the left index finger controls the *q*_*2*_ joint (moving the finger up decreases the angle), the right index finger controls the *q*_*3*_ joint (moving the finger up increases the angle) and the right middle finger controls the *q*_*1*_ joint (moving the finger up decreases the angle; cf. Fig 9A and 9B). Within a session, this mapping was fixed.

For the participants that started in condition *H*_*1*_ (half the participants), these mappings were randomized in both sessions. Each participant that started in condition *H*_*2*_ was assigned the same mappings in the same order as a previous participant who started in condition *H*_*1*_. Thereby, we reduced possible biases introduced by the random finger-to-joint mapping, as each mapping was assigned twice, once in each condition.

Each session comprised of 24 training blocks. Each training block was followed by a test phase (Fig 9C). Participants started a block by positioning their fingers in the respective home posture (*H*_*1*_ or *H*_*2*_; see Task and Fig 9B). The corresponding finger posture was indicated in green on the screen (Fig 9C). After taking this posture, the white target ellipse appeared and participants saw it continually deform for 80 seconds. This deformation of the target ellipse corresponds to a trajectory connecting all training targets in a random order (Fig 9C). That is, five seconds of deformation correspond to a straight path from one target to the next. Reach-endpoint feedback (black ellipse) corresponding to participant’s actions was flashed for just two frames every second. Whenever the target ellipse reached one of the training targets, a score between 0 and 100 was displayed on the screen for the following five second. The score was calculated by *score* = max(0,(1−*d*)^2^) ⋅ 100 where *d* is the distance between target and reach-endpoint in solution coordinates (*x* and *y*). Participants were instructed to track the white ellipse with the black ellipse by moving the three fingers (LI, RI, RM) up and down and to thereby maximize the score. They had no information about the kinematics of the underlying three DoF arm.

During the test phase, participants received visual feedback about the finger positions in the real world but not about the reach-endpoint position in goal space (Fig 9C, bottom left). White ellipses corresponding to the nine test targets in goal space (cf. Task) were displayed in a random order for as long as it took participants to respond. Participants were instructed to take a posture that they believe corresponds to the target shape and then submit their response by stepping on a foot pedal with the right foot. They received a score for their response right after submitting (same calculation as during training, but accumulating over test trials). At the end of the test phase, an overview screen displayed the participant’s progress (sum of training and test scores for all previous blocks). Pressing the foot pedal again initiated the next block (i.e., taking home posture to start training).

### Data analysis

The following variables were analysed to assess differences in test performance between the two conditions and across time. In 1.4% of the cases, the motion sensor did not register the position of all three fingers. These data points (175 in total) were discarded and treated as missing data in all the analyses below.

For most of the variables, we tested for differences using two-way rmANOVAs to test for the effects of the factors Time (block number or bin-of-block number) and Condition (*H*_*1*_, *H*_*2*_). For the factor Condition, we decided to use the finger mapping as grouping variable, which was counter-balanced across participants (cf. Procedure) rather than the participant identity. Especially for synergy learning, finger mapping had a strong biasing effect (Fig 8 left), which seemed more important to consider in the analysis than participant identity (both was not possible). The ANOVA tables are reported as supporting tables S1 Table, S2 Table, S3 Table and S4 Table. Partial eta-squared ($\eta_{P}^{2}$) was used as a measure of statistical effect size as it is established in the literature, even if generalized eta-squared ($\eta_{G}^{2}$) is regarded by some as preferable [46].

#### Performance

Performance was evaluated as the metric distance *d* between a given target and the reach-endpoint in goal space (*x* and *y*). The average error $\bar{d}$ across test targets was compared across participants and conditions using a two-way rmANOVA with factors: Time (block number: 1–24) and Condition (*H*_*1*_, *H*_*2*_). The full statistical results are in S1 Table. We did not model the learning as exponential decay because the learning dynamics varied drastically between participants and included both, initial increases of and sudden drops in error (see S1 Fig).

#### Postures in motor space (q_1_, q_2_, q_3_)

For data analysis in motor space, we ‘unfolded’ the joint angles *q*_*i*_. That is, if a finger was elevated by a distance that exceeds an angular position of *q*_*i*_ *=* 2π or goes below *q*_*i*_ = 0, we kept the original values even if they were outside the range of [0, 2π] as also the underlying finger movements were not circular. The interpretation in the goal space, however, was circular. That is, the effects of joint angles *q*_*i*_ = -π, *q*_*i*_ = π and *q*_*i*_ = 3π (different locations in motor space) are identical in the goal space (reach-endpoint position).

To analyse the formation of synergies (motor organization), we performed a Principal Component Analysis (PCA) of the postures in motor space (*q*_*1*_, *q*_*2*_, *q*_*3*_*)* used to perform reaches to the nine test targets during test blocks. The results from three consecutive test blocks were pooled, i.e., each PCA was performed on 27 data points (3 blocks x 9 test targets) with no pre-processing. The analysis was performed for each participant and condition using the function pca.m of the MATLAB Statistics Toolbox. We investigated the *relative variance explained* in a solution (P1: How many principal components are necessary to explain behaviour?), the *location* of learned solutions (P2: Which posture forms the centre of a solution and how far are they from the home postures?), the *relative variability of DoFs* (P3: Which DoFs are used more in which PCs and how does that relate to task success?) and the *absolute variability of DoFs* (What is the spread of *q*_*i*_ values used?).

The change in variance explained by PCs across time (synergy formation, P1) was analysed as the matched-sample contrast between the variance explained in the first time-bin (blocks 1–3) and the last time bin (blocks 22–24). Sign tests were performed for the pooled results across conditions (*H*_*1*_, *H*_*2*_) for all three synergies (PC1, PC2, PC3).

To test whether the home posture biases the location of solutions in motor space (P2), we projected the average posture in each block onto a line connecting *H*_*1*_ and *H*_*2*_. The location on this line was compared between conditions using two-way rmANOVA with factors: Time (bin-of-three-blocks number: 1–8) and Condition (*H*_*1*_, *H*_*2*_). The full statistical test results are in S2 Table.

Our analysis of the relative use of DoFs was concentrated on the absolute value of *q*_*2*_ in PC1, as we had clear predictions as to how this value would behave (P3, see main text). To test for differences between conditions, we analysed the contrast between conditions *H*_*1*_ and *H*_*2*_. We first tested for effects of the factor: Time using a Friedman ANOVA. As there was no evidence for a difference between blocks (p = 0.943; full ANOVA table in S4 Table), a Wilcoxon sign rank test was performed on the across block median.

The absolute variability of DoFs was analysed using a two-way rmANOVA with factors: Time (bin-of-three-blocks number: 1–8) and Condition (*H*_*1*_, *H*_*2*_). The full statistical test results are in S3 Table.

#### Finger positions (humans only)

To test for constraints imposed by human morphology, we analysed finger elevation *el* in physical space (an elevation of *el =* 0 cm implies that a finger is held level). We pooled the results from all participants (same fingers across participants, but different assignments to *q*_*1*_, *q*_*2*_ and *q*_*3*_) and calculated a PCA for each three consecutive learning blocks to test for systematic finger co-activation patterns. This was compared to an analogous PCA of the pooled results in terms of the experimental task’s motor space (*q*_*1*_, *q*_*2*_ and *q*_*3*_ postures, different finger movements assigned across participants) to test whether variance across participant is better explained by human morphology or by task constraints.

## Supporting information

S1 Dataset A .zip file with all data reported in the manuscript. The 220 .json files contain the data (20 for the participants, 200 for simulation runs), the text file legend_json.txt explains the data format.
(ZIP)

S1 FigIndividual learning curves.Mean distance $\bar{d}$ across training blocks. Odd numbered participants started in Condition *H*_*1*_, even numbered participants started in Condition *H*_*2*_.(TIFF)

S2 Fig Reach performance for different test targets (agent population). For each individual, the reach-endpoints during the last test block are plotted. The ellipses are the across agent covariance ellipses.
(TIFF)

S3 Fig Reach performance for different test targets (human population). For each individual, the reach-endpoints for the median postures across the last three test blocks are plotted. The ellipses are the across participant covariance ellipses.
(TIFF)

S4 Fig Average reach performance for different test targets across time. Lines represent across individual mean. Left: condition H1, right: condition H2. Top: simulated agents, bottom: human participants. Solid lines represent interpolation targets, dotted lines represent extrapolation targets. The brightness represents distance from the home posture (the brighter the closer).
(TIFF)

S1 TableANOVA table for two-way repeated-measures ANOVA (rmANOVA) to test for differences in the mean distance to the targets (error) with factors: Time (block number 1–24) and Condition (H1, H2).SSq. Stands for the sum of squares, DF for Degrees of Freedom, Mean Sq. for the Mean Squared Error, F for the F statistics, p-value for the probability that the null hypothesis (sample means are equal) is true given the observed values and $\eta_{P}^{2}$ stands for partial eta-squared (effect size).(DOCX)

S2 TableANOVA table for two-way repeated-measures ANOVA (rmANOVA) to test for differences in the relative distance of solutions to the two home positions with factors: Time (8 levels) and Condition (H1, H2).SSq. Stands for the sum of squares, DF for Degrees of Freedom, Mean Sq. for the Mean Squared Error, F for the F statistics, p-value for the probability that the null hypothesis (sample means are equal) is true given the observed values and $\eta_{P}^{2}$ stands for partial eta-squared (effect size).(DOCX)

S3 TableANOVA table for two-way repeated-measures ANOVA (rmANOVA) to test for differences in the summed variance in motor space with factors: Time (8 levels) and Condition (*H_1_*, *H_2_*).SSq. Stands for the sum of squares, DF for Degrees of Freedom, Mean Sq. for the Mean Squared Error, F for the F statistics, p-value for the probability that the null hypothesis (sample means are equal) is true given the observed values and $\eta_{P}^{2}$ stands for partial eta-squared (effect size).(DOCX)

S4 Table Friedman ANOVA to test for differences in the relative use of the *q2* joint in the first Principal Component (contrast conditions H1 and H2) across Time (8 levels). (DOCX)

S1 Video A clip of the task as seen through the eyes of a participant (training phase). (MP4)
