# Supplementary figures and images for: Goal-related feedback guides motor exploration and redundancy resolution in human motor skill acquisition
Source: PLoS Comput Biol. 2019 Mar 5;15(3):e1006676. doi: 10.1371/journal.pcbi.1006676 (PMC6420027; doi:10.1371/journal.pcbi.1006676)

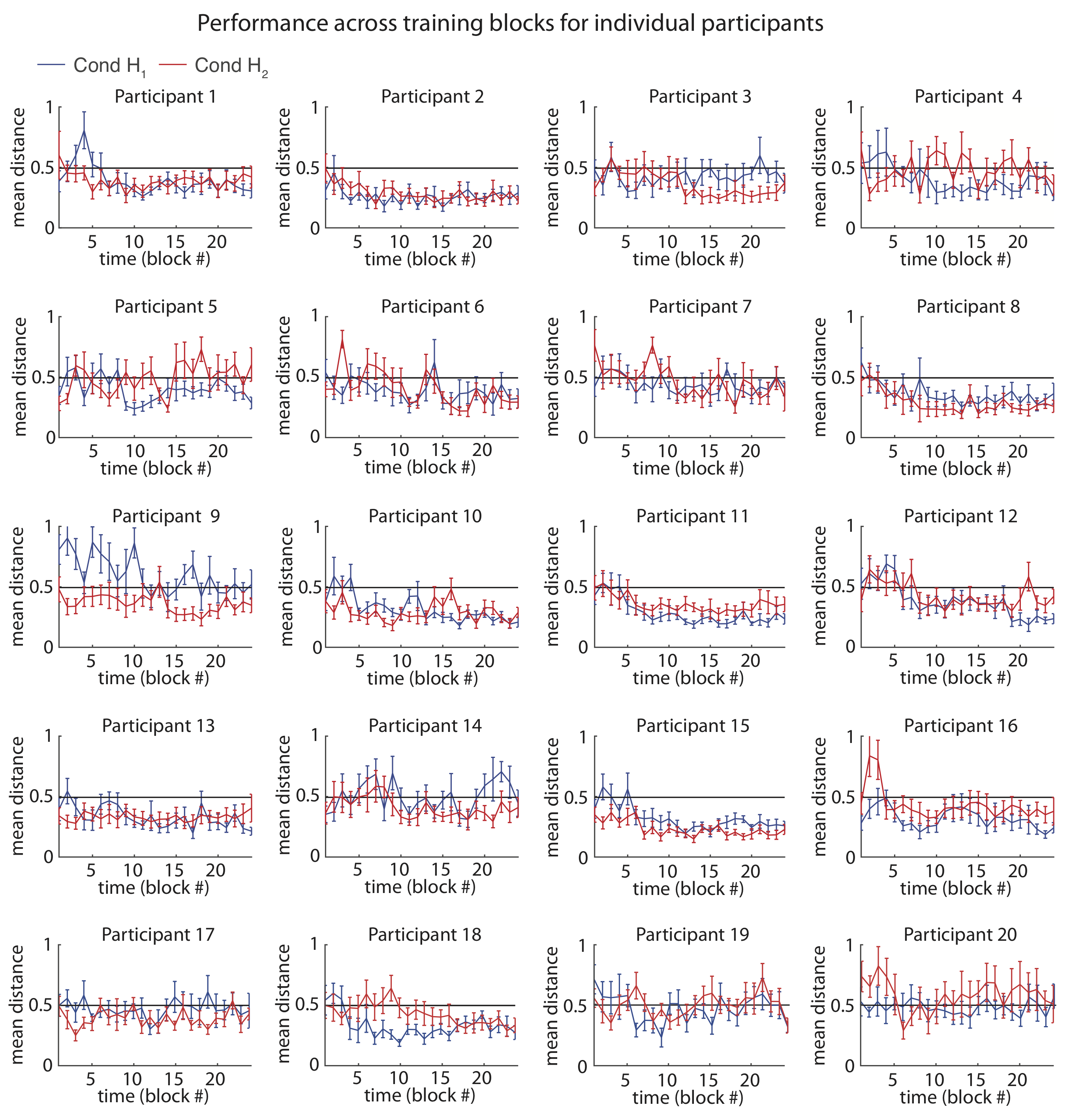

Supplement: S1 Fig — Mean distance d¯ across training blocks. Odd numbered participants started in Condition H1, even numbered participants started in Condition H2. (TIFF) [file pcbi.1006676.s002.tiff]

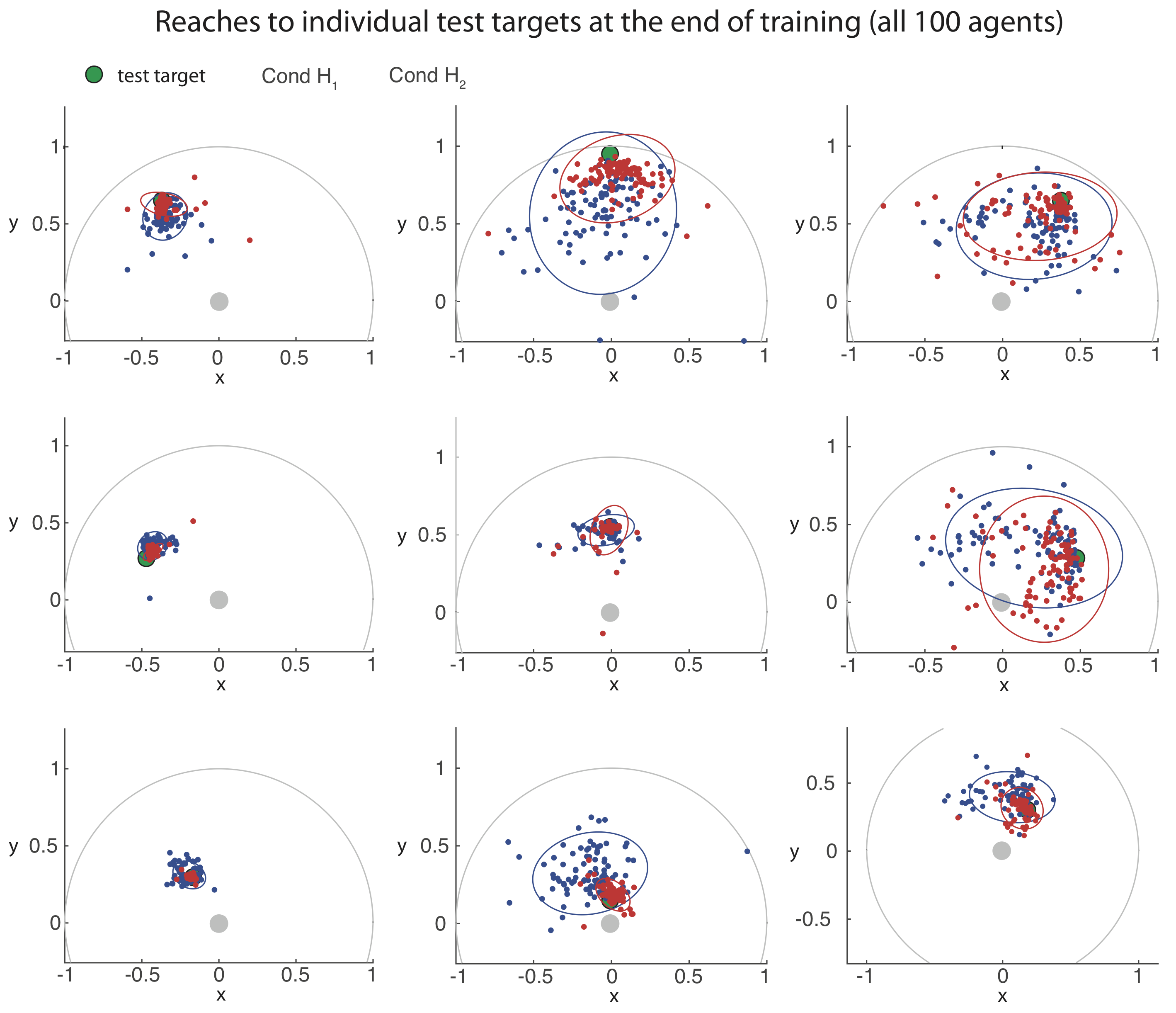

Supplement: S2 Fig — For each individual, the reach-endpoints during the last test block are plotted. The ellipses are the across agent covariance ellipses. (TIFF) [file pcbi.1006676.s003.tiff]

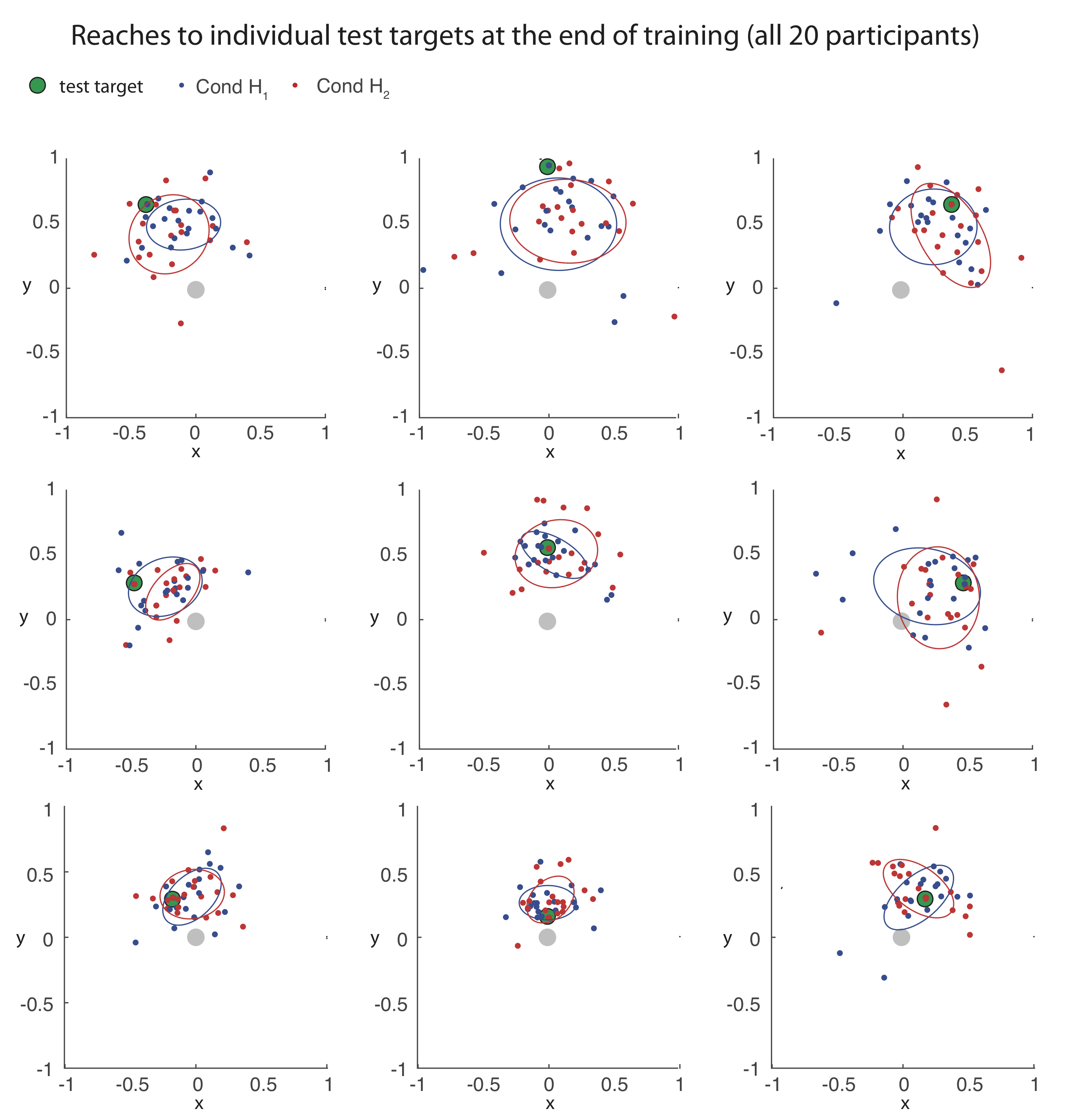

Supplement: S3 Fig — For each individual, the reach-endpoints for the median postures across the last three test blocks are plotted. The ellipses are the across participant covariance ellipses. (TIFF) [file pcbi.1006676.s004.tiff]

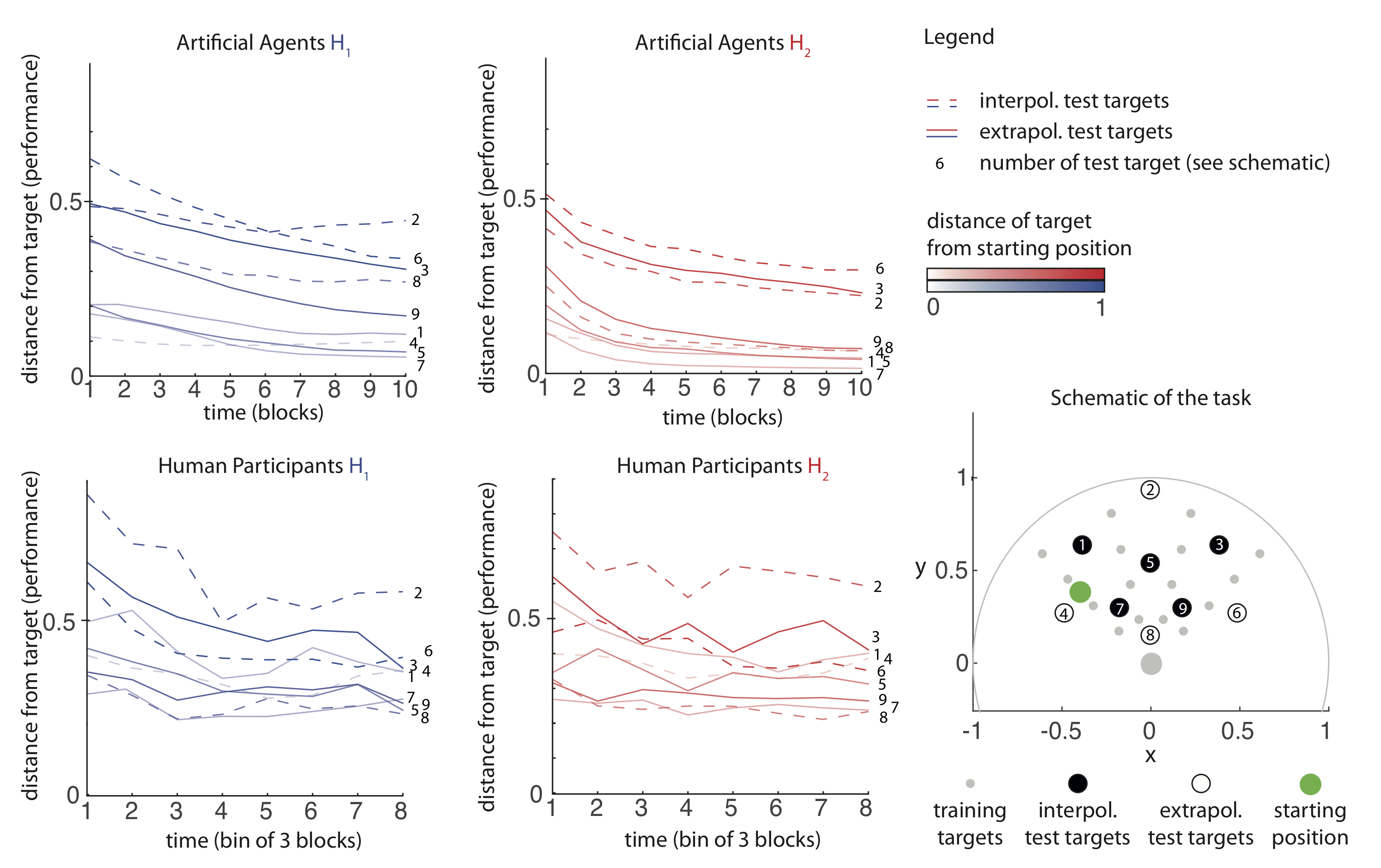

Supplement: S4 Fig — Lines represent across individual mean. Left: condition H1, right: condition H2. Top: simulated agents, bottom: human participants. Solid lines represent interpolation targets, dotted lines represent extrapolation targets. The brightness represents distance from the home posture (the brighter the closer). (TIFF) [file pcbi.1006676.s005.tiff]
