# Supplementary material for: Goal-related feedback guides motor exploration and redundancy resolution in human motor skill acquisition
Source: PLoS Comput Biol. 2019 Mar 5;15(3):e1006676. doi: 10.1371/journal.pcbi.1006676 (PMC6420027; doi:10.1371/journal.pcbi.1006676)
Supplement: S1 Table — SSq. Stands for the sum of squares, DF for Degrees of Freedom, Mean Sq. for the Mean Squared Error, F for the F statistics, p-value for the probability that the null hypothesis (sample means are equal) is true given the observed values and ηP2 stands for partial eta-squared (effect size). (DOCX) [file pcbi.1006676.s006.docx]

**Source SSq. DF Mean Sq. F p-value** $\boldsymbol{\eta}_{\boldsymbol{P}}^{\boldsymbol{2}}$

Subject 3.376 19 0.178 1.2 0.355

Condition 0.001 1 0.001 0.01 0.44 0

Time 1.962 23 0.085 10.9 0 0,364

Subject x Condition 2.838 19 0.149 19.1 0

Subject x Time 3.427 437 0.008 1.0 0.480

Condition x Time 0.174 23 0.008 1.0 0.507 0,049

Error 3.410 437 0.008

Total 15.189 959
