# Supplementary material for: Goal-related feedback guides motor exploration and redundancy resolution in human motor skill acquisition
Source: PLoS Comput Biol. 2019 Mar 5;15(3):e1006676. doi: 10.1371/journal.pcbi.1006676 (PMC6420027; doi:10.1371/journal.pcbi.1006676)
Supplement: S2 Table — SSq. Stands for the sum of squares, DF for Degrees of Freedom, Mean Sq. for the Mean Squared Error, F for the F statistics, p-value for the probability that the null hypothesis (sample means are equal) is true given the observed values and ηP2 stands for partial eta-squared (effect size). (DOCX) [file pcbi.1006676.s007.docx]

**Source SSq. DF Mean Sq. F p-value** $\boldsymbol{\eta}_{\boldsymbol{P}}^{\boldsymbol{2}}$

Subject 135.08 19 7.11 1.4 0.326

Condition 23.34 1 23.34 4.56 0.046 0.193

Time 0.84 7 0.12 0.44 0.875 0.023

Subject x Condition 97.21 19 5.12 16.06 0

Subject x Time 36.12 133 0.27 0.85 0.821

Condition x Time 1.42 7 0.20 0.64 0.725 0.032

Error 42.38 133 0.32

Total 336.36 319
