# Supplementary material for: Goal-related feedback guides motor exploration and redundancy resolution in human motor skill acquisition
Source: PLoS Comput Biol. 2019 Mar 5;15(3):e1006676. doi: 10.1371/journal.pcbi.1006676 (PMC6420027; doi:10.1371/journal.pcbi.1006676)
Supplement: S3 Table — SSq. Stands for the sum of squares, DF for Degrees of Freedom, Mean Sq. for the Mean Squared Error, F for the F statistics, p-value for the probability that the null hypothesis (sample means are equal) is true given the observed values and ηP2 stands for partial eta-squared (effect size). (DOCX) [file pcbi.1006676.s008.docx]

**Source SSq. DF Mean Sq. F p-value** $\boldsymbol{\eta}_{\boldsymbol{P}}^{\boldsymbol{2}}$

Subject 209.26 19 11.01 0.5 0.919

Condition 1.991 1 1.99 0.1 0.762 0.005

Time 23.08 7 3.30 2.4 0.024 0.112

Subject x Condition 398.83 19 20.99 18.1 0

Subject x Time 182.91 133 1.38 1.2 0.164

Condition x Time 7.87 7 1.12 1.0 0.457 0.049

Error 154.34 133 1.16

Total 978.27 319
