# Supplementary material for: Goal-related feedback guides motor exploration and redundancy resolution in human motor skill acquisition
Source: PLoS Comput Biol. 2019 Mar 5;15(3):e1006676. doi: 10.1371/journal.pcbi.1006676 (PMC6420027; doi:10.1371/journal.pcbi.1006676)
Supplement: S4 Table — (DOCX) [file pcbi.1006676.s009.docx]

**Source SSq. DF Mean Sq. Chi-sq p-value**

16.5 7 2.36 2.75 0.907

Error 823.5 133 6.19

Total 840 159
